# Persistent Low-Level Variants in a Subset of Viral Genes Are Highly Predictive of Poor Outcome in Immunocompromised Patients With Cytomegalovirus Infection

**DOI:** 10.1093/infdis/jiae001

**Published:** 2024-01-05

**Authors:** Cristina Venturini, Julia M Colston, Oscar Charles, Anastasia Lankina, Timothy Best, Claire Atkinson, Calum Forrest, Charlotte A Williams, Kanchan Rao, Austen Worth, Doug Thorburn, Mark Harber, Paul Griffiths, Judith Breuer

**Affiliations:** Infection, Immunity and Inflammation, Institute of Child Health, University College London (UCL); North Bristol National Health Service (NHS) Trust, University Hospitals Bristol and Weston NHS Foundation Trust, Bristol; Infection, Immunity and Inflammation, Institute of Child Health, University College London (UCL); Infection, Immunity and Inflammation, Institute of Child Health, University College London (UCL); Virology Department, Great Ormond Street Hospital for Children NHS Foundation Trust; Applied Science, London South Bank University; Division of Infection and Immunity, Institute for Immunity and Transplantation, UCL; Division of Infection and Immunity, Institute for Immunity and Transplantation, UCL; Department of Genetics and Genomic Medicine, Great Ormond Street Institute of Child Health, UCL; Department of Bone Marrow Transplant; Department of Immunology, Great Ormond Street Hospital for Children NHS Foundation Trust, London; Department of Nephrology, Royal Free London NHS Foundation Trust, London, United Kingdom; Department of Nephrology, Royal Free London NHS Foundation Trust, London, United Kingdom; Division of Infection and Immunity, Institute for Immunity and Transplantation, UCL; Infection, Immunity and Inflammation, Institute of Child Health, University College London (UCL); Virology Department, Great Ormond Street Hospital for Children NHS Foundation Trust

**Keywords:** cytomegalovirus infection, genomics, viral signature, transplant

## Abstract

**Background:**

Human cytomegalovirus (HCMV) is the most common and serious opportunistic infection after solid organ and hematopoietic stem cell transplantation. In this study, we used whole-genome HCMV data to investigate viral factors associated with the clinical outcome.

**Methods:**

We sequenced HCMV samples from 16 immunocompromised pediatric patients with persistent viremia. Eight of the 16 patients died of complications due to HCMV infection. We also sequenced samples from 35 infected solid organ adult recipients, of whom 1 died with HCMV infection.

**Results:**

We showed that samples from both groups have fixed variants at resistance sites and mixed infections. Next-generation sequencing also revealed nonfixed variants at resistance sites in most of the patients who died (6/9). A machine learning approach identified 10 genes with nonfixed variants in these patients. These genes formed a viral signature that discriminated patients with HCMV infection who died from those who survived with high accuracy (area under the curve = 0.96). Lymphocyte numbers for a subset of patients showed no recovery posttransplant in the patients who died.

**Conclusions:**

We hypothesize that the viral signature identified in this study may be a useful biomarker for poor response to antiviral drug treatment and indirectly for poor T-cell function, potentially identifying early those patients requiring nonpharmacological interventions.

## Teaser

In this study, we explore the complex relationship between cytomegalovirus infections and clinical outcomes in transplant recipients and propose strategies for monitoring viral dynamics to optimize treatment and prevent failure to respond to antiviral therapies.

Human cytomegalovirus (HCMV; human herpesvirus 5) is a member of the Betaherpesvirinae subfamily with a worldwide seroprevalence of between 18% and 100% [1, 2]. HCMV is usually a benign viral infection in immunocompetent individuals; however, it is a significant cause of morbidity and mortality in immunosuppressed patients [3, 4]. Therefore, strategies for prevention as well as treatment are of paramount importance for transplant clinical success. Several therapies exist for prophylaxis, preemptive therapy, and/or treatment of HCMV [5]. Treatment with ganciclovir (GCV), foscarnet (FOS), cidofovir, or letermovir has improved outcomes [6–8], although late resistance often occurs [9]. Despite excellent outcomes for most hematopoietic stem cell transplant (HSCT) and solid organ transplant (SOT) recipients, severe life-threatening HCMV disease can develop in approximately 20% to 50% of cases [7, 10]. Next-generation sequencing (NGS) has associated the presence of fixed drug mutations and mixed infections with poorer outcomes [11–14]. To further investigate the pathogenesis of life-threatening HCMV in immunocompromised patients, we analyzed the viral populations of 16 children with persistent HCMV viremia. We also analyzed a cohort (n = 35) of immunocompromised adults with persistent HCMV.

## METHODS

### Sample Collection and Ethics

#### Great Ormond Street Hospital Samples

Whole blood samples were stored at Great Ormond Street Hospital for Children (GOSH) at −80°C. These residual samples were collected as part of the standard clinical care at GOSH and subsequently approved for research use through the University College London (UCL) Partners Infection DNA Bank by the NRES Committee London Fulham (REC reference: 12/LO/1089) and West Midlands Black Country Research Ethics Committee (REC reference: 18/WM/0186). All samples were anonymized. Informed patient consent was not required.

#### Royal Free London Samples

Samples were collected as part of the Wellcome collaborative grant (204870/Z/16/Z UKRI) “Analysis of Cytomegalovirus Pathogenesis in Solid Organ Transplant Patients” approved by the NRES Committee London Queens Square Ethics Committee (REC reference 17/LO/0916).

#### Sequencing

Nucleic acid was enriched using custom baits and sequenced as previously described [11, 12, 15] (Supplementary Materials).

### Data Availability

Raw sequencing data for HCMV have been deposited in the European Nucleotide Archive (project accession number PRJEB12814 and PRJEB55677 for GOSH patients and PRJEB55701 for SOT patients from the Wellcome Trust cohort at Royal Free hospital).

### Statistical Analysis

Bioinformatics processing and statistical analyses are described in the Supplementary Materials. The viral signature score model and data, along with a script for calculating scores for new samples, can be found in the GitHub repository: https://github.com/ucl-pathgenomics/HCMV_ViralSignature.

## RESULTS

### Patients’ Characteristics

We analyzed 16 retrospectively identified pediatric patients from GOSH with primary immunodeficiency disorders (PIDs), HSCTs, or SOTs (Supplementary Database 1). All had HCMV viremia persisting with ≤0.5 log reduction despite antiviral treatment for 21 days or longer, which has been defined as refractory [16]. No patients received prophylaxis against HCMV, although all HSCT recipients received standard acyclovir prophylaxis against alpha-herpesviruses. Preemptive antiviral treatment for HCMV was initiated at first detection in the PIDs when viremia exceeded 1000 IU/mL in the HSCT recipients and 3000 IU/mL in the SOT recipients. First-line therapy was ganciclovir in the SOT recipients and children with PIDs and foscarnet in the HSCT recipients. We stratified patients into 2 groups: a poor outcome group, defined as those who died with HCMV viremia (n = 8), and a good outcome group, defined as patients who cleared their HCMV (n = 8). We analyzed an additional cohort of 35 adult SOT recipients with persistent HCMV viremia. Patients underwent either liver or kidney transplant at the Royal Free Hospital. Preemptive antiviral treatment was started with valganciclovir and ganciclovir on the first positive PCR (>200 ge/mL) and stopped on the second negative PCR (<200 ge/mL). Subsequent viremia >3000 ge/mL was treated again with valganciclovir and ganciclovir. In this cohort, only 1 patient (liver recipient) died following persistent HCMV viremia.

### Sequencing Data

We analyzed a total of 141 HCMV sequences: 59 samples from 16 immunocompromised children (1–9 samples per patient) collected over time and 82 samples from 35 immunocompromised adults. All samples selected for this study had an average sequencing depth of unique reads of ≥10 reads/nucleotide, and ≥95% coverage of the strain Merlin genome [17]. The average sequencing depth in these 2 cohorts ranged from 10× to 1407× (after removing duplicates) (Supplementary Database 1).

### Multiple HCMV Strain Infection and Outcomes

**Figure 1.**
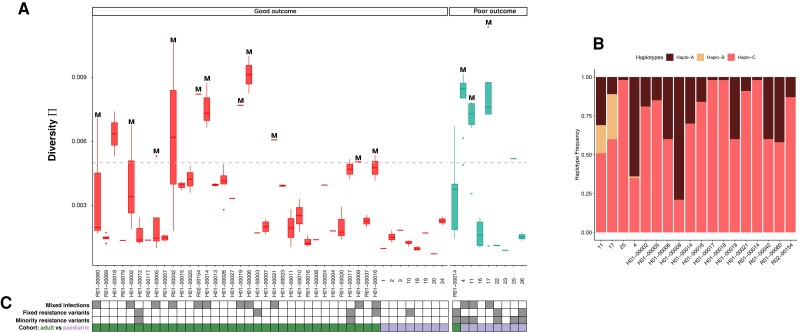
*A*, Diversity values for all patients. Each patient (x-axis) had longitudinal samples (box plots) showing diversity values (y-axis). Patients with good clinical outcomes are represented in red and those with poor outcomes are in turquoise. Dashed gray line represents the chosen cutoff of 0.005 for investigating mixed infections. Mixed infections are labeled with “M.” *B*, Reconstructed haplotype abundances for 18 patients suspected from their diversity scores of having mixed infections. Haplotypes were reconstructed for each patient; here, for representative purposes, we only show 1 sample for each patient. Minor haplotypes occurring at <5% were discounted [19]. *C*, Summary heatmap showing patients with mixed infection, patients with known fixed or minority variants at resistance sites, and cohort information (green for the adult cohort and violet for the pediatric cohort).

To investigate the presence of multiple viral strains, we first calculated genome-wide within-host diversity (π) for each sample [12] (Figure 1*A*) and reconstructed haplotypes for suspected mixed infections (Figure 1*B* and Supplementary Figure 1) [18]. We identified a total of 14 mixed infections in both cohorts. Taken together, mixed infections were not predictive of clinical outcome (26% of patients with good outcomes with multiple strains versus 33% with poor outcomes; χ^2^ = 0.19, *P* = .66).

### Drug Resistance Mutations and Minority Variants

We investigated mutations in the UL97 (serine/threonine protein kinase) and the UL54 (DNA polymerase) genes, which are the targets of the anti-HCMV drugs used here (GCV, FOS) [20].

In the pediatric cohort, 2 patients (P22 and P26) showed fixed mutations at resistance sites (ie, present in most of the viral population) (Table 1). In the adult cohort, we identified fixed variants at resistance sites in 5 patients (R01-00014, H01-00017, H01-00016, H01-00012, H01-00003). Overall, we did not find any difference between patients based on the presence of fixed variants at resistance sites (5/42 patients [11.9%] in the good outcome group and 3/9 [33.5%] in the poor outcome group; χ^2^ = 2.57, *P* = .19).

**Table 1.** All Detected Drug Resistance Mutations (Fold-Change ≥2)

Clinical Outcome	Patient	Fixed		Minority Variants	
		UL54	UL97	UL54	UL97
Poor	P22	K513N^a^, Q578L	M460I	E756D, Q578L^b^, A809V, L802M^a^	…
	P4	…	…	D588N^a^, V715M	C592G, T409M, M460I
	P11	…	…	T813S, V715M	…
	P25	…	…	E756D/Q, N408K, Q578H, L773V, A834P, G841A, A987G	C603W, H520Q, M460I^a^, A594P
	P26	…	M460V	…	A594V
	R01-00014	L545S	M460I	L545S^b^	M460V
	3 patients (P16, P17, and P23) with poor clinical outcome did not show any resistance mutations				
Good	H01-00017	N408K	M460I	N408K^b^	…
	H01-00016	…	L595S	…	…
	H01-00012	…	C603W	…	C603W^b^
	H01-00003	…	L595S	…	…
	P10	L501I^c^	G598D^c^	…	…
	37 patients with good clinical outcome did not show any resistance mutations				

^a^Detected by Sanger sequencing and next-generation sequencing.

^b^Variants rising to fixation.

^c^Detected only by Sanger sequencing.

We then investigate the presence of minority variants (MVs) (ie, present in the minority of the viral population). To take into account the presence of artefactual variability in sequence samples, only MVs occurring at a frequency of ≥2% [21] and minimum variant depth ≥5 reads were considered. In both cohorts, NGS sequencing revealed low-frequency GCV and FOS resistance mutations in 6 of 9 of the patients with poor outcome, 2 of whom also had mixed infections, and 2 of 42 in the good prognosis group, none of whom had mixed infections (χ^2^ = 21.47, *P* = .00001; Table 1). Most variants occurred at frequencies <15% (median frequency, 13.45) (Supplementary Figure 2). In all patients who died, low-frequency resistance mutations persisted in multiple longitudinal samples (when available), with the majority failing to rise to fixation (Supplementary Figure 2). In contrast, drug resistance MVs present in the 2 patients who did well (adult cohort) did rise to fixation in later samples (Supplementary Figure 2).

We did not identify any resistance mutations in our sequenced samples for P10 (n = 6, including days 5, 11, 14, 18, and days 75 and 174 postadmission), but Sanger sequencing detected 2 resistance mutations: L501I in UL54 only on day 18 of treatment (day 43 postadmission) and G598D in UL97 only on day 81 postadmission (treatment day 56).

### Minority Variants in All Viral Genes

To better investigate the dynamics of HCMV genome variation, we expanded our analysis to include MVs in the whole genome in single infections (Supplementary Figure 3). Interestingly, nonsynonymous (NS) HCMV MVs were not confined to antiviral resistance sites but were distributed randomly across the HCMV genome in both the pediatric and adult cohorts (Supplementary Figure 4).

To investigate whether specific regions were enriched for NS MVs, we directly compared and ranked HCMV genes that discriminated patients who died from patients who survived using machine learning methods. We combined the 2 cohorts to increase statistical power. The gene selection process identified 10 genes (K score > 8, *P* < .005, adjusted *P* < .5) (Supplementary Figure 5, Supplementary Table 1) that showed more within-host variability in the poor outcome group compared to the good outcome group. No gene showed the opposite trend.

The variable genes in the patients with poor outcome included the polymerase gene (UL54) and the serine/threonine protein kinase gene (UL97), already known for drug resistance. In addition, we identified genes coding for glycoproteins (envelope gp such as UL74, gO, and UL75, gH; immediate early gp, UL37; membrane gp, UL7), membrane proteins UL121 and UL8, and the genes coding for the uncharacterized proteins UL20 and UL11, the latter of which plays a role altering host immune response by modulating T-cell function.

We focused on NS MVs as these gave better discrimination between poor and good outcome groups than NS and synonymous mutations combined, for all genes, bar UL11, UL7, and UL97 (Supplementary Table 1).

### Viral Signature in HCMV Samples From Patients With Poor Clinical Outcome

**Figure 2.**
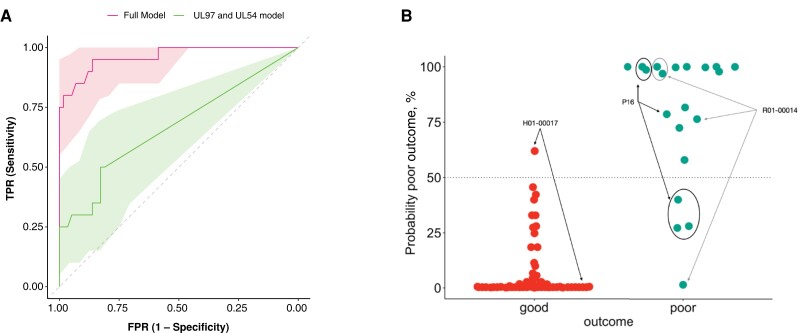
*A*, Receiver operating characteristic (ROC) curves with 95% confidence intervals for 2 predictive models discriminating between samples from patients who died and survivors. Area under the curve (AUC) for the full model (including minority variants in the 10 candidate genes) was 0.96 (red ROC curve). AUC for the drug resistance gene model (including genes UL54 and UL97) was 0.65 (green ROC curve). *B*, Estimated probabilities for each sample in the 2 groups (red for survivors, turquoise for patients who died) to be classified as a patient from the poor outcome group. Arrows and circle indicate patients with at least 1 sample that was misclassified by the model. Abbreviations: FPR, false positive rate; TRP, true positive rate.

We assessed the power of our 10-gene viral signature to predict poor clinical outcomes in HCMV samples. Two models were employed: 1 considering the presence/absence of MVs in all 10 genes and another focusing on known resistance genes (UL54 and UL97). Generalized logistic models (glm) were used, and receiver operating characteristic curves with area under the curve (AUC) were employed for model evaluation. The full model achieved an AUC of 0.96, significantly outperforming the model with only resistance genes (*P* < .001, analysis of variance; Figure 2*A*). Probability estimates generated by the 10-gene glm model indicated the likelihood of unfavorable clinical outcomes for each observation (Figure 2*B*).

Only 5 samples from 3 patients were misclassified by the full model (Supplementary Table 2, Figure 2*B*). In the adult cohort, 1 sample from patient H01-00017 who survived was classified as “poor outcome.” A second sample (45 days later) had, however, a probability of 0% of being poor outcome. This patient was 1 of only 2 who survived with multiple resistance mutations, 1 fixed and another that rose to fixation in the second sample. A second patient (R01-00014) from the adult cohort had 1 sample misclassified (1/4). This patient died and most samples showed high probabilities of poor outcome. Patient P16 from the pediatric cohort showed a more complex picture over time. This patient died and most samples (4/7) showed concordant probabilities >50% of poor outcome. The samples with high probabilities were interspersed with samples showing low probabilities of poor outcome. All of these were taken before HSCT in the first 20 days after admission. We found that these discrepancies were not due to lower viral loads or samples’ quality (Supplementary Table 2), but they probably reflect the complex HCMV dynamics and the need for repeated testing for accurate results.

We also assessed the predictive power of the signature including mixed infections. The full model including mixed infections had a high predictive power (AUC = 0.91), albeit lower than the model with only single infections (AUC = 0.96), likely due to the difficulty in assembly and calling MVs where multiple strains are present (Supplementary Figure 6).

To validate whether our signature was specific for immunocompromised individuals following antiviral treatment, we examined sequences (n = 29, from amniotic fluid) from congenital HCMV infections publicly available (Supplementary Table 3). Although congenital HCMV infections had higher variability than samples from immunocompromised patients (Supplementary Figures 7 and 8), we found fewer MVs in the 10 genes of the viral signature than in samples from patients who died.

### Viral Signature Over Time

**Figure 3.**
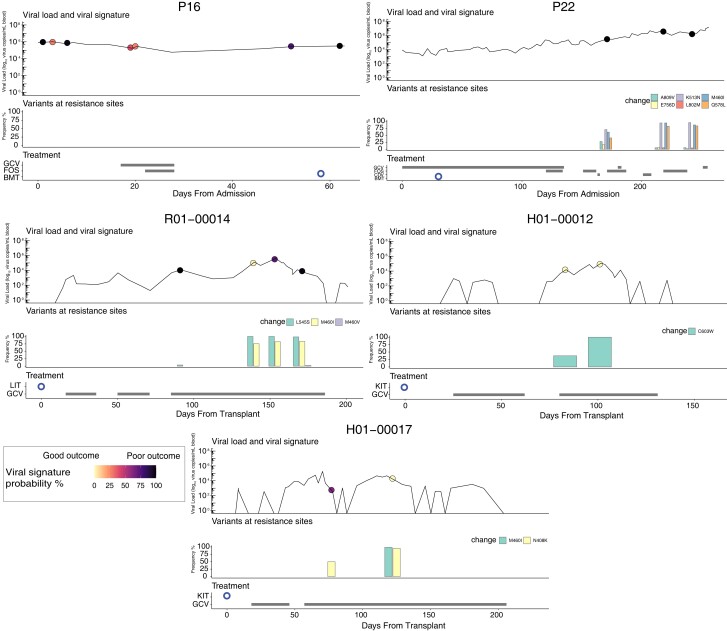
Viremia, antiviral therapy and transplantation, variants at resistance sites, and viral signature probability in patients with longitudinal samples and low-level resistance mutations, including patients 16, 22, and R01-00014 from the poor outcome group and H01-00012 and H01-00017 from the good outcome group. Dots indicate samples sequenced and are colored based on viral signature probabilities (from yellow [good outcome] to black [poor outcome]). Black rectangles indicate antiviral treatment, and the blue circles show the time of transplant (hematopoietic stem cell transplant for patient 16, liver for patient R01-00014, thymus for patient 22, and kidney for patients H01-00012 and H01-00017). Abbreviations: BMT, bone marrow transplant; CDV, cidofovir; FOS, foscarnet; GCV, ganciclovir; KIT, kidney transplant; LIT, liver transplant.

To determine how early MVs in the 10 sentinel genes can be used to predict a potentially poor outcome, we plotted the probability of being in the poor outcome group for 3 patients who died and had longitudinal samples (Figure 3). We also plotted longitudinal data for the 2 patients who recovered and had MVs at resistance sites. We did not have samples earlier during HCMV infection for patients 22 and R01-00014. However, samples taken at days 171 (from admission) and 91 (from transplant), respectively (62 and 109 days before death), were positive for the predictive signature. In patient P16 the signature was present as early as 9 days after HSCT.

### Biological Significance of the MVs

Most of the HCMV genome is under purifying selection [22], presenting on average a greater proportion of synonymous changes compared to NS and stop codons. Surprisingly, 5 of the 10 genes in our viral signature (UL54, UL20, UL121, UL97, and UL74) reversed this trend with greater NS vs synonymous MVs (Table 2). In the genes, NS variants tended to cluster closer together than expected by chance, suggesting a functional role. In addition, most of the MVs (63%) mapped to HCMV variable loci identified comparing GenBank sequences. A higher overlap was observed for hypervariable genes (eg, UL74 [23]) compared with drug resistance genes (eg, UL54) (Table 2).

**Table 2.** Biological Features of the 10 Human Cytomegalovirus Genes Under Investigation in Patients Who Died

Gene	Description	% of NS MVs in Variable Sites	NS–S	NS–S Control	Do NS Variants Cluster Together?	Epitopes
UL54	DNA polymerase catalytic subunit	0	33–13	10–22	*P* = 3.4 × 10^-05^	MLLDKEQM**A**LK; L**E**NGVTHRF; NHGAGG**T**AAVSYQGA
UL20	Uncharacterized	92%	57–42	13–21	*P* = 3.6 × 10^-03^	MLG**IR**AMLVMLDYYW; SST**EGN**WSVTNLTES; MLL**PR**QYTL; FMDY**V**ILTP**L**AVLTC
UL11	Plays a role in the modulation of host immune response by modulating T-cell function	44%	34–39 2 stop codon	20–17	*P* = 7.6 × 10^-04^	CYYVYV**TQ**NGTLPTT
UL8	Membrane protein	83%	47–47	23–35	*P* = 6.5 × 10^-01^	**S** SD**WV**TLGTSA**S**LL**R**
UL37	Immediate early glycoprotein	63%	52–52	26–27	*P* = 2.8 × 10^-04^	No epitope
UL121	Membrane protein	66%	11–7	10–10	*P* = 1.4 × 10^-01^	VCLILSFSIV**T**AALW; ISL**V**TPLTINATLRL; SCTHPYVISL**V**TPLT
UL75	Envelope glycoprotein gH	100%	25–63	8–18	*P* = 9 × 10^-04^	FPDATV**P**ATV; K**A**QLNRHSYLKDSDFLDAA; RQTEKHELLVLVKK**A**QLNRH; HELLVLVKK**A**QL; YLLSHLPSQRYGADAASEALD**P**HAFHLLLNTYGRPIRFLRENTTQC; A**A**SE**A**LD**P**HAFHLLLNTYGR; LD**K**AFHLLL; YL**L**SHL**P**SQRYGA**D**A**A**SE**A**LDPHAFHLLLNTYGRPIRFLRENTTQC
UL7	CEACAM1-like protein; plays a role in modulating the host immune response	78%	20–33	13–20	*P* = 3.5 × 10^-02^	STPYVGLS**LS**CAANQ
UL97	Serine/threonine protein kinase	11%	15–11	10–2	*P* = 1.1 × 10^-02^	No epitope
UL74	Envelope glycoprotein gO	92%	64–59	22–36	*P* = 2.9 × 10^-05^	LLFLD**E**IRNFSL**RS**P; TMRK**L**KRKQALVKEQ; SFY**L**VNAMSRNLFRV

The table shows the % of NS variants mapping to human cytomegalovirus variable sites, the number of NS vs S MVs found, *P* values indicating whether the NS MVs clustered significantly closer than by chance, and known and predicted T-cell epitopes from the IEDB database that MVs mapped to (the position of the MV in the epitope is shown in boldfaced, underlined text).

Abbreviations: MV, minority variant; NS, nonsynchronous; S, synchronous.

The clustering of variable residues is a feature of epitopes for which plasticity provides advantages in the face of host immunity. We identified known and predicted T-cell epitopes (IEDB database) overlapping with amino acid changes in patients who died in 8 of 10 genes (including the 5 genes with NS > synonymous).

### Lymphocyte Counts in Patients With Poor Clinical Outcomes

**Figure 4.**
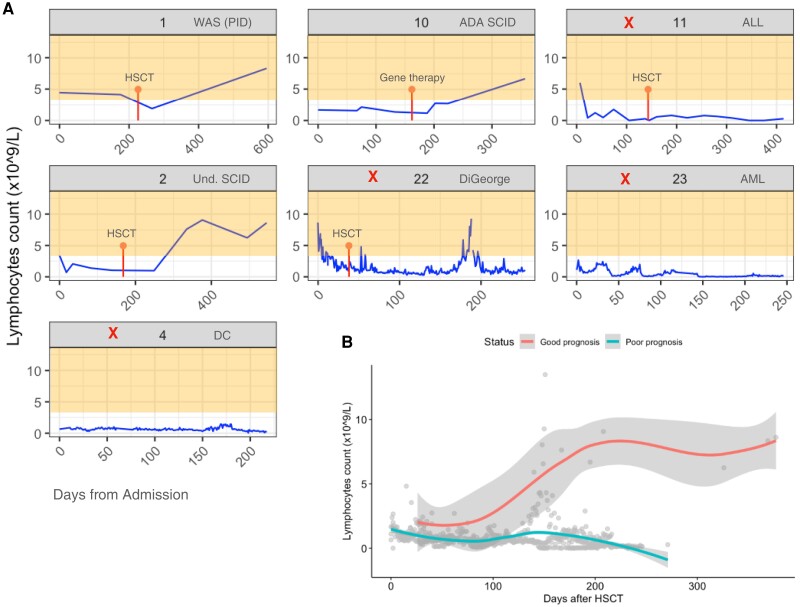
*A*, Lymphocyte count (per microliter of blood) over time in a subset of patients at Great Ormond Street Hospital for Children. Time of hematopoietic stem cell transplant (HSCT) or gene therapy is shown in red. In orange we indicate the healthy lymphocyte count range for children (3–13 cells/μL). Patients with poor outcomes are indicated with a red X. *B*, Trend lines (smoothed local regression line using loess) for lymphocyte count for and poor outcome groups after HSCT or gene therapy (or after admission for patients who did not have HSCT/gene therapy). The gray area represents 95% confidence interval. Abbreviations: ADA, Adenosine deaminase deficiency; ALL, Acute lymphoblastic leukemia; AML, Acute myeloid leukemia; DC, Dyskeratosis congenita; HSCT, hematopoietic stem cell transplant; PID, Primary immunodeficiency; SCID, Severe Combined Immunodeficiency; Und., Undetermined; WAS, Wiskott-Aldrich syndrome.

The finding that MVs are significantly more likely to occur in regions predicted to be immunogenic led us to explore how immunity might relate to the presence of these MVs. Lymphocyte counts were available from a subset of pediatric patients (n = 7: P1, P2, P4, P10, P11, P22, P23) (Figure 4*A*). In patients P1 and P2 (who received HSCTs) and patient P10 (who received gene therapy), lymphocyte counts recovered quickly after treatment (Figure 4*A*). In contrast, patients P4, P11, P22, and P23, who died, showed no recovery of lymphocyte count after HSCT. Lymphocyte counts were persistently low in both groups just after HSCT or gene therapy and started to increase at day 100 after transplant. Linear mixed effect modeling showed a significant difference in the counts over time (*P* < .001; Figure 4*B*) with significant differences in the final lymphocyte counts (good outcome: median lymphocyte count, 8.34 [95% confidence interval [CI], 6.69–8.34; poor outcome: median lymphocyte count, 0.275 [95% CI, .14–1.10]).

Analyzing the SOT adult cohort separately (subset n = 10 patients), as lymphocyte counts change with age, patient R01-00014 who died also showed persistently lower lymphocyte counts for months after receiving liver transplant as compared with the rest of the SOT cohort (Supplementary Figures 9 and 10) (last time point before death for R01-00014 was 0.22; the median in the rest of the SOT patients was 1.61 [95% CI, .68–1.61]).

## DISCUSSION

Cytomegalovirus (HCMV) is the most common cause of infection following bone marrow and solid organ transplantations [7, 24]. The mechanisms by which HCMV infection influences transplant outcome are not known [7], but drug-resistant HCMV strains and infection with multiple strains have been associated with increased morbidity and mortality [11–14].

To investigate viral factors influencing transplant outcome, we sequenced longitudinal HCMV samples from 16 immunocompromised children with persistent viremia. Half of this cohort died of HCMV complications. We also sequenced samples from 35 adult SOT recipients in which only 1 patient died following persistent levels of HCMV viremia.

Multiple-strain infections are common in immunocompromised individuals; in our study, we identified a slightly higher percentage of mixed infections in patients who died (33% vs 26%), but the difference was not significant.

The use of antiviral drugs in the treatment of HCMV disease perturbs the viral population, selecting for drug resistance variants. About 25% of the patients analyzed in this study showed resistance mutations at various frequencies in the DNA polymerase UL54 and the protein kinase UL97, which are the major drug targets. Fixed mutations were present in patients independent of the outcome. In contrast, MVs were almost exclusively present in samples from patients who died. Interestingly, low-frequency resistant variants detected in 2 patients who survived quickly rose to fixation, whereas those detected in 3 patients who died persisted at low frequencies in the longitudinal samples.

Compared to traditional sequencing, NGS can detect resistance mutations at low frequency at high resolution, enabling the detection of evolving virus populations in immunocompromised individuals selected under antiviral treatment [11, 17, 25, 26]. Thus, the finding of MVs at drug resistance sites should trigger repeat testing to better define the phenotype as well as to identify early resistance mutations that may become fixed and require treatment change.

These data and previous observations confirm that HCMV is highly stable at the consensus level in immunocompromised patients with very few substitutions observed over time in single-strain infections (0–25 substitutions) [12, 17, 27]. To further investigate the greater within-host viral variation in some patients, we used a machine learning approach to attempt to discriminate between patients who died and those who survived. Using only samples from single infections, we identified the presence of MVs in 1 or more of 10 genes, including UL54 and UL97, as discriminatory between the 2 groups. Notwithstanding the opportunistic nature of the samples available, we were able to detect this signature on average 84 days before death and <100 days posttransplant, and in all cases the signature was present in the first available sample. Interestingly, we identified the signature even in samples without resistance mutations from patients who died (P16, P23, P17). In addition, our signature seems to be distinctive for samples in immunocompromised patients as we could not find it in samples from congenital infections.

Only 2 genes of the signature were involved in drug resistance. Half of the genes included in the signature had a higher proportion of NS variants than expected by purifying selection [22], mapped to known HCMV variable genes, and these loci clustered more than expected by chance. These results suggest positive selection, a hallmark of immune epitopes (Table 2), and indeed, in 7 of 10 of the signature protein genes MVs mapped to known HCMV T-cell epitopes. There might be several reasons why these variants remain at low frequency. Although variation at the consensus level is rare due to the proofreading activity of the viral DNA polymerase [28], 1 possibility is that low-level variation in these epitopes occurs normally but is cleared by functional T-cell immunity. Variants are unlikely to confer increased fitness, rising to fixation only in circumstances where they enable evasion of prevailing immunity. In the absence of functional T-cell immunity, as in the patients who died described here, we postulate that variants arising in epitopes can persist at low frequencies long enough to allow detection by NGS sequencing. In addition, there is evidence that GCV resistance mutations are not evenly distributed in different cell compartments [29, 30], and the presence of low-level virus subpopulations with antiviral resistance may represent virus confined in certain cell types.

Taken together, the data hint at the possibility that dysregulated immunity contributes to the accumulation of MVs. Early studies showed that recovery of CD8^+^ T cells and CD4^+^ T cells is a positive predictor for prevention of mortality in HCMV disease [31–33]. Restoration of HCMV-specific cytotoxic T lymphocytes (CTL) response (class I MHC-restricted specific CD8^+^ CTL) may require an extended time after transplant in some patients, and such patients are at increased risk of developing severe HCMV disease. In our study, we were not able to obtain a measurement of T-cell function, largely because the peripheral blood lymphocyte subset counts were too low for the assays used. Instead, we analyzed lymphocyte counts as a proxy for lymphocyte function in a subset of 7 children for whom data were available. None of the 4 patients (3 post-HSCT and 1 with PID) who died had measurable lymphocyte counts, and all harbored viral variants in the 10 genes as described above. In contrast, 3 subjects with a good outcome (2 post-HSCT and 1 after gene therapy) for whom we had data showed good lymphocyte count recovery. Thus, the detection of persistent low-frequency HCMV mutations may be a biomarker of poor immune reconstitution and consequent poor outcome of HCMV infection.

Although fatal HCMV disease is less common in SOTs, it is interesting that patient R01-00014 (from the adult cohort) who died with disseminated HCMV showed a similar signature to the pediatric patients, suggesting that similar processes may underlie fatal HCMV disease irrespective of transplant type.

In this opportunistically collected sample set, we did not always have samples early on in HCMV infection. Notwithstanding, the viral signature was present in all cases in the first sample tested. In all cases the signature was detected <100 days after transplant, that is, before T-cell recovery is expected, thus providing a potential early biomarker for failure of engraftment and poor outcome of HCMV infection. Since low-frequency resistance mutations that later rise to fixation can occur in the good prognosis group, repeated testing to demonstrate persistent MVs is likely to increase specificity.

This study comprises initial observations drawn from a relatively small cohort, particularly within the poor outcome group, with samples from children collected opportunistically during routine clinical care at GOSH. Given the opportunistic nature of sample collection, these observations lack the structure of a preplanned study. To validate and extend our findings, it will be crucial to conduct prospective studies with a larger and more diverse patient population, incorporating longitudinal sampling. The importance of preplanned sampling cannot be emphasized enough, as it enables the incorporation of technical and clinical replicates, enhancing the robustness and generalizability of our results. A further limitation is that the biological basis for these observations is not known, although we speculate as to a possible explanation.

Despite the availability of effective antivirals, HCMV remains a serious infection, particularly in the context of immunocompromised individuals. Routine use of NGS in refractory patients could potentially detect significant resistance at earlier time points. At the same time, repeated detection of MVs may prove to be a useful biomarker for poor response to drug treatment alone and identify patients for whom nonpharmaceutical rescue therapies may be needed.

## Supplementary Data

Supplementary materials are available at *The Journal of Infectious Diseases* online (http://jid.oxfordjournals.org/). Supplementary materials consist of data provided by the author that are published to benefit the reader. The posted materials are not copyedited. The contents of all supplementary data are the sole responsibility of the authors. Questions or messages regarding errors should be addressed to the author.

## Supplementary Material

jiae001_Supplementary_Data

## Notes

***Acknowledgments.*** We are grateful for the excellent help from the Pathogen Genomics stream of University College London (UCL) Genomics.

***Financial support*.** This work was supported by the Wellcome Trust (grant number 204870/Z/16/Z to C. V., C. A., and C. F.). J. B. receives funding from the University College London Hospitals Biomedical Research Centre. A.L. is supported by the BBSRC London Interdisciplinary Doctoral Programme (LIDo). O.C. was supported by a MRC UKRI PhD studentship (MR/N013867/1).
