# Supplementary material for: Persistent Low-Level Variants in a Subset of Viral Genes Are Highly Predictive of Poor Outcome in Immunocompromised Patients With Cytomegalovirus Infection
Source: J Infect Dis. 2024 Jan 5;230(2):e427–36. doi: 10.1093/infdis/jiae001 (PMC11326829; doi:10.1093/infdis/jiae001)
Supplement: jiae001_Supplementary_Data [file jiae001_supplementary_data.zip › Supplementary_Database_1.docx]

| **ID** | **Sample ID** | **HCMV Load (viral copies/ml blood)** | **Sample Date** | **Average read depth (after duplicates removal)** | **Within-host diversity (π)** | **Coverage HCMV (NC.006273) genome%** | **ENA short read accession number** | **Project ENA** | **Single/Mixed** | **Patient condition** | **Duration of viraemia** | **Days treatment** | **Peak** | **Outcome** | **Anti-virals** | **Transplant/Gene therapy** | **More notes about clinical outcome** | **Cohort** | **Previously published?** |
| --- | --- | --- | --- | --- | --- | --- | --- | --- | --- | --- | --- | --- | --- | --- | --- | --- | --- | --- | --- |
| 1 | p1_2015_04_29 | 203794 | 29\4\2015 | 35.55 | 0.00099 | 97.9 | ERS13443603 | PRJEB55677 | Single | Wiskott-Aldrich syndrome | 36 (300 days detected by PCR at any level) | 66 | 505906 | good | ACV, GCV, VGC | SCT | Well after gene therapy | Paediatric | No |
| 2 | p2_2015_08_03 | 283740 | 3\8\2015 | 35.64 | 0.00184 | 99.45 | ERS13443604 | PRJEB55677 | Single | Undefined severe combined immunodeficiency | 26 (281 days detected by PCR at any levels) | 285 | 583630 | good | ACV, FOS, GCV | SCT | Well after SCT. Has nephrotic syndrome unrelated to SCID | Paediatric | No |
|  | p2_2015_08_06 | 217728 | 6\8\2015 | 61.27 | 0.0016 | 99.94 | ERS13443605 | PRJEB55677 |  |  |  |  |  |  |  |  |  |  |  |
|  | p2_2015_08_12 | 583630 | 12\8\2015 | 112.48 | 0.00144 | 100 | ERS13443606 | PRJEB55677 |  |  |  |  |  |  |  |  |  |  |  |
|  | p2_2015_08_20 | 48473 | 20\8\2015 | 44.88 | 0.00125 | 98.26 | ERS13443607 | PRJEB55677 |  |  |  |  |  |  |  |  |  |  |  |
| 3 | p3_14_03_28 | 2134470 | 28\3\2014 | 39.64 | 0.00184 | 97.97 | ERR1279034 | PRJEB12814 | Single | B-acute lymphoblastic leukemia | 28 ( >80 days detected by PCR at any levels)) | 30 | 2134470 | good | FOS, GCV | SCT | Well after SCT | Paediatric | Yes - patient A in Houldcroft et al., 2016 |
| 4 | p4_2014_08_21 | 2231870 | 21\8\2014 | 71.42 | 0.00803 | 99.71 | ERR1279046 | PRJEB12814 | Mixed | Dyskeratosis congenita | 200 | 180 | 65611500 | poor | FOS, GCV, CDV, MBV, LEF, ART, CMV-IVIG |  | Did not have SCT. Died of CMV colitis and CMV pneumonities | Paediatric | Yes - patient A in Cudini et al., 2019; patient B in Houldcroft et al., 2016 |
|  | p4_2014_08_28 | 3472940 | 28\8\2014 | 71.71 | 0.00819 | 99.44 | ERR1279026 | PRJEB12814 |  |  |  |  |  |  |  |  |  |  |  |
|  | p4_2014_09_01 | 3428740 | 1\9\2014 | 80.08 | 0.00876 | 99.8 | ERR1279027 | PRJEB12814 |  |  |  |  |  |  |  |  |  |  |  |
|  | p4_2014_09_04 | 4332230 | 4\9\2014 | 159.87 | 0.00917 | 99.34 | ERR1279048 | PRJEB12814 |  |  |  |  |  |  |  |  |  |  |  |
|  | p4_2014_09_22 | 1250500 | 22\9\2014 | 128.74 | 0.00868 | 99.64 | ERR1279050 | PRJEB12814 |  |  |  |  |  |  |  |  |  |  |  |
|  | p4_2014_09_25 | 7690670 | 25\9\2014 | 371.63 | 0.00872 | 100 | ERR1279028 | PRJEB12814 |  |  |  |  |  |  |  |  |  |  |  |
|  | p4_2014_10_23 | 418339 | 23\10\2014 | 71.85 | 0.00846 | 98.77 | ERR1279055 | PRJEB12814 |  |  |  |  |  |  |  |  |  |  |  |
|  | p4_2014_10_30 | 587500 | 30\10\2014 | 44.32 | 0.00615 | 98.36 | ERR1279029 | PRJEB12814 |  |  |  |  |  |  |  |  |  |  |  |
|  | p4_2014_11_10 | 11076300 | 10\11\2014 | 575.79 | 0.0049 | 99.86 | ERR1279057 | PRJEB12814 |  |  |  |  |  |  |  |  |  |  |  |
| 10 | p10_2014_11_25 | 3965090 | 25\11\2014 | 326.08 | 0.00123 | 99.73 | ERR1279059 | PRJEB12814 | Single | ADA SCID | 45 (>180 days detected by PCR at any levels) | 65 | 3965090 | good | ACV, FOS, GCV, CDV, palivizumab | Gene therapy | Well after gene therapy | Paediatric | Yes - patient E in Cudini et al., 2019; patient H in Houldcroft et al., 2016 |
|  | p10_2014_11_26 | 1075780 | 26\11\2014 | 67.68 | 0.00123 | 99.25 | ERR1279062 | PRJEB12814 |  |  |  |  |  |  |  |  |  |  |  |
|  | p10_2014_11_27 | 3453580 | 27\11\2014 | 49.23 | 0.00136 | 98.95 | ERR1279036 | PRJEB12814 |  |  |  |  |  |  |  |  |  |  |  |
|  | p10_2014_12_01 | 1591210 | 1\12\2014 | 31.28 | 0.00135 | 97.79 | ERR1279037 | PRJEB12814 |  |  |  |  |  |  |  |  |  |  |  |
|  | p10_2015_01_26 | 137686 | 26\1\2015 | 21.86 | 0.00108 | 98.74 | ERS13443594 | PRJEB55677 |  |  |  |  |  |  |  |  |  |  |  |
|  | p10_2015_05_05 | 84959 | 5\5\2015 | 39.16 | 0.00121 | 98.37 | ERS13443595 | PRJEB55677 |  |  |  |  |  |  |  |  |  |  |  |
| 11 | p11_2015_10_22 | 164163 | 22\10\2015 | 19.9 | 0.00773 | 97.55 | ERS13443596 | PRJEB55677 | Mixed | Acute Lymphoblastic leukaemia | 274 | 234 | 1257520 | poor | GCV, FOS, VGC, ACV | SCT | Marrow transplant, died due to gram negative sepsis after CART cell therapy | Paediatric | No |
|  | p11_2015_10_26 | 45141 | 26\10\2015 | 53.45 | 0.00693 | 98.41 | ERS13443597 | PRJEB55677 |  |  |  |  |  |  |  |  |  |  |  |
|  | p11_2015_10_29 | 219830 | 29\10\2015 | 104.58 | 0.00781 | 98.56 | ERS13443598 | PRJEB55677 |  |  |  |  |  |  |  |  |  |  |  |
|  | p11_2015_11_02 | 323667 | 2\11\2015 | 46.68 | 0.00783 | 98.04 | ERS13443599 | PRJEB55677 |  |  |  |  |  |  |  |  |  |  |  |
|  | p11_2015_12_30 | 250280 | 30\12\2015 | 124.04 | 0.0073 | 99.73 | ERS13443600 | PRJEB55677 |  |  |  |  |  |  |  |  |  |  |  |
|  | p11_2016_07_11 | 874748 | 11\7\2016 | 146.41 | 0.00664 | 99.74 | ERS13443601 | PRJEB55677 |  |  |  |  |  |  |  |  |  |  |  |
|  | p11_2016_07_22 | 1257520 | 22\7\2016 | 64.02 | 0.00355 | 97.83 | ERS13443602 | PRJEB55677 |  |  |  |  |  |  |  |  |  |  |  |
| 16 | p16_2015_09_04 | 803282 | 4\9\2015 | 136.03 | 0.00232 | 98.66 | ERS13432764 | PRJEB55677 | Single | RAG2 SCID | 62 | 11 | 1016020 | poor | GCV, FOS | SCT | Presented with CMV pneumonitis and died after SCT of CMV pneumonitis | Paediatric | No |
|  | p16_2015_09_07 | 1016020 | 7\9\2015 | 67.32 | 0.00243 | 98.18 | ERS13432765 | PRJEB55677 |  |  |  |  |  |  |  |  |  |  |  |
|  | p16_2015_09_10 | 553418 | 10\9\2015 | 98.45 | 0.00219 | 98.53 | ERS13432766 | PRJEB55677 |  |  |  |  |  |  |  |  |  |  |  |
|  | p16_2015_09_14 | 472109 | 14\9\2015 | 17.04 | 0.00199 | 96.42 | ERS13432768 | PRJEB55677 |  |  |  |  |  |  |  |  |  |  |  |
|  | p16_2015_09_23 | 174148 | 23\9\2015 | 28.88 | 0.00113 | 99.23 | ERS13432770 | PRJEB55677 |  |  |  |  |  |  |  |  |  |  |  |
|  | p16_2015_09_24 | 281838 | 24\9\2015 | 61.92 | 0.00102 | 98.37 | ERS13432771 | PRJEB55677 |  |  |  |  |  |  |  |  |  |  |  |
|  | p16_2015_10_26 | 282830 | 26\10\2015 | 58.88 | 0.00107 | 98.27 | ERS13432774 | PRJEB55677 |  |  |  |  |  |  |  |  |  |  |  |
|  | p16_2015_11_05 | 320073 | 5\11\2015 | 90.41 | 0.00124 | 98.35 | ERS13432775 | PRJEB55677 |  |  |  |  |  |  |  |  |  |  |  |
| 17 | p17_2015_09_16 | 217700 | 16\9\2015 | 14.76 | 0.00876 | 97.39 | ERS13432776 | PRJEB55677 | Mixed | Acute Lymphoblastic leukaemia | 62 | 137 | 250965 | poor | GCV, CDV | SCT | PBSCT transplant died after thrombotic microangiopathy after SCT | Paediatric | No |
|  | p17_2015_09_24 | 152943 | 24\9\2015 | 22.98 | 0.0076 | 98.22 | ERS13432778 | PRJEB55677 |  |  |  |  |  |  |  |  |  |  |  |
|  | p17_2015_09_28 | 147419 | 28\9\2015 | 23.18 | 0.00728 | 98.49 | ERS13432779 | PRJEB55677 |  |  |  |  |  |  |  |  |  |  |  |
|  | p17_2015_09_30 | 126599 | 30\9\2015 | 17.69 | 0.0011 | 97.62 | ERS13432780 | PRJEB55677 |  |  |  |  |  |  |  |  |  |  |  |
|  | p17_2015_11_17 | 118151 | 17\11\2015 | 23.84 | 0.01136 | 98.61 | ERS13432781 | PRJEB55677 |  |  |  |  |  |  |  |  |  |  |  |
| 18 | p18_2015_10_10 | 140345 | 10\10\2015 | 54.96 | 0.00101 | 98.41 | ERS13432782 | PRJEB55677 | Single | Undefined immunodeficiency | 90 (142 days detected by PCR at any levels) | 55 | 2014670 | good | GCV, VGC |  | Noted to have absent B and NK cells, CD3 lymphopenic and low IgG despite SCIG therapy.  Multiple chest issues, including CMV pneumonitis and ongoing persistent CMV viraemia | Paediatric | No |
|  | p18_2015_10_12 | 151809 | 12\10\2015 | 35.04 | 0.00091 | 98.05 | ERS13432783 | PRJEB55677 |  |  |  |  |  |  |  |  |  |  |  |
|  | p18_2016_01_27 | 215920 | 27\1\2016 | 41.43 | 0.00113 | 98.72 | ERS13432784 | PRJEB55677 |  |  |  |  |  |  |  |  |  |  |  |
|  | p18_2016_02_09 | 35311 | 9\2\2016 | 10.38 | 0.00091 | 95.66 | ERS13432787 | PRJEB55677 |  |  |  |  |  |  |  |  |  |  |  |
| 19 | p19_2015_09_11 | 174872 | 11\9\2015 | 10.15 | 0.00171 | 95.84 | ERS13432790 | PRJEB55677 | Single | Undefined immunodeficiency | 81 (> 2 y detected by PCR at any levels)) | 293 | 3795840 | good | ACV, FOS, GCV, VGC | SCT | Chronic lung diseases. EBV driven lymphoproliferative disease. Well after SCT | Paediatric | No |
| 20 | p20_2016_02_02 | 52855 | 2\2\2016 | 11.62 | 0.00074 | 97.17 | ERS13432797 | PRJEB55677 | Single | Chronic granulomatous disease (p40 deficiency) | 23 | 15 | 52855 | good | VGC | SOT | LRD renal transplant (father) Nov 2015. CMV viraemia, acute T-cell mediated rejection. Received methylpred, plasma exchange, rituximab, IVIG. | Paediatric | No |
|  | p20_2016_02_09 | 45973 | 9\2\2016 | 10.06 | 0.00073 | 97.74 | ERS13432799 | PRJEB55677 |  |  |  |  |  |  |  |  |  |  |  |
| 22 | p22_171 | 333831 | 171 days after admission (2013) | 22.1 | 0.00112 | 98.16 | ERR1279035 | PRJEB12814 | Single | DiGeorge syndrome | 157 (> 2 y detected by PCR at any levels) | >200 | 16721700 | poor | FOS, GCV, CDV, palivizumab | Thymus transplant | Died after thymic transplant due to chronic lung disease. Persistently high levels CMV viraemia | Paediatric | Yes - patient F in Cudini et al., 2019; patient I in Houldcroft et al., 2016 |
|  | p22_218 | 5114408 | 218 days after admission (2013) | 354.5 | 0.00115 | 99.6 | ERR1279043 | PRJEB12814 |  |  |  |  |  |  |  |  |  |  |  |
|  | p22_242 | 3528590 | 242 days after admission (2013) | 410.67 | 0.00112 | 99.83 | ERR1279044 | PRJEB12814 |  |  |  |  |  |  |  |  |  |  |  |
| 23 | p23_49 | 988020 | 49 days after admission (2013) | 45.55 | 0.00089 | 98.7 | ERR1279040 | PRJEB12814 | Single | Acute myeloid leukaemia | 57 (>90 detected by PCR at any levels) | 43 | 11728700 | poor | ACV, FOS | SCT | Died after SCT due to disease relapse | Paediatric | Yes - patient G in Cudini et al., 2019; patient J in Houldcroft et al., 2016 |
|  | p23_170 | 1228210 | 170 days after admission (2013) | 270.37 | 0.00091 | 99.41 | ERR1279041 | PRJEB12814 |  |  |  |  |  |  |  |  |  |  |  |
|  | p23_217 | 7189820 | 217 days after admission (2013) | 611 | 0.0009 | 99.62 | ERR1279042 | PRJEB12814 |  |  |  |  |  |  |  |  |  |  |  |
| 24 | p24_49 | 393192 | 26/11/2014 | 37.13 | 0.00247 | 98.23 | ERR1279032 | PRJEB12814 | Single | Heart transplant | 12 (32 detected by PCR at any levels) | 39 | 393192 | good | GCV, V-GCV | SOT | On-going treatment | Paediatric | Yes - patient H in Cudini et al., 2019; patient K in Houldcroft et al., 2016 |
|  | p24_241 | 104165 | 29/11/2014 | 10.54 | 0.00208 | 97.64 | ERR1279031 | PRJEB12814 |  |  |  |  |  |  |  |  |  |  |  |
| 25 | p25_2018_11_19 | 1112646 | 19\11\2018 | 370.88 | 0.00519 | 99.74 | ERS13432802 | PRJEB55677 | Single | Acute Lymphoblastic leukaemia | 90 (98 detected by PCR at any levels) | NA | 9160000 | poor | GCV, FOS, VGC, ACV |  | Death | Paediatric | No |
| 26 | p26_2019_11_07 | 22 (CT values) | 7\11\2019 | 100.54 | 0.00173 | 99.32 | ERS13432803 | PRJEB55677 | Single | DiGeorge syndrome | 27 | 28 | >20000000 | poor | CDV, FOS, GCV, |  | Too unwell to receive thymus transplant due to disseminated CMV disease. Transferred back home for palliation | Paediatric | No |
|  | p26_2019_11_21 | 21 (CT values) | 21\11\2019 | 774.95 | 0.00133 | 99.84 | ERS13432804 | PRJEB55677 |  |  |  |  |  |  |  |  |  |  |  |
| H01-00002 | H01-00002-160909-B | 3966 | 09/09/2016 | 24.71 | 0.00188 | 98.04 | ERS13431175 | PRJEB55701 | Mixed | D+R- liver recipient | 75 | 86 | 7181 | good | V-GCV or GCV on 1st positive >200 ge/ml. Rx stopped on 2nd negative CMV PCR <200 ge/ml. Subsequent viraemia >3000 ge/ml V-GCV or GCV starts | SOT |  | Adult | No |
|  | H01-00002-161020-B | 7202 | 20/10/2016 | 19.54 | 0.00341 | 98.13 | ERS13431176 | PRJEB55701 |  |  |  |  |  |  |  |  |  |  |  |
|  | H01-00002-161205-B | 6420 | 05/12/2016 | 37.65 | 0.00677 | 99.32 | ERS13431177 | PRJEB55701 |  |  |  |  |  |  |  |  |  |  |  |
| H01-00003 | H01-00003-170116-B | 13731 | 16/01/2017 | 26.45 | 0.00171 | 99.44 | ERS13431179 | PRJEB55701 | Single | D+R- kidney recipient | 90 | 46 | 7202 | good | V-GCV or GCV on 1st positive >200 ge/ml. Rx stopped on 2nd negative CMV PCR <200 ge/ml. Subsequent viraemia >3000 ge/ml V-GCV or GCV starts | SOT |  | Adult | No |
| H01-00004 | H01-00004-161114-B | 34452 | 14/11/2016 | 110.92 | 0.00181 | 99.76 | ERS13431180 | PRJEB55701 | Single | D+R- liver recipient | 37 | 50 | 34452 | good | V-GCV or GCV on 1st positive >200 ge/ml. Rx stopped on 2nd negative CMV PCR <200 ge/ml. Subsequent viraemia >3000 ge/ml V-GCV or GCV starts | SOT |  | Adult | No |
| H01-00005 | H01-00005-170212-B | 14964 | 12/02/2017 | 13.69 | 0.00146 | 97.59 | ERS13431182 | PRJEB55701 | Mixed | D+R- kidney recipient | 111 | 125 | 1794110 | good | V-GCV or GCV on 1st positive >200 ge/ml. Rx stopped on 2nd negative CMV PCR <200 ge/ml. Subsequent viraemia >3000 ge/ml V-GCV or GCV starts | SOT |  | Adult | No |
|  | H01-00005-170330-B | 11924 | 30/03/2017 | 55.3 | 0.00531 | 99.45 | ERS13431183 | PRJEB55701 |  |  |  |  |  |  |  |  |  |  |  |
|  | H01-00005-170404-B | 5383 | 04/04/2017 | 21.42 | 0.001 | 98.41 | ERS13431184 | PRJEB55701 |  |  |  |  |  |  |  |  |  |  |  |
|  | H01-00005-170105-B | 35154 | 05/01/2017 | 41.05 | 0.00133 | 98.48 | ERS13431185 | PRJEB55701 |  |  |  |  |  |  |  |  |  |  |  |
|  | H01-00005-170126-B | 612005 | 26/01/2017 | 453.79 | 0.00242 | 99.92 | ERS13431187 | PRJEB55701 |  |  |  |  |  |  |  |  |  |  |  |
| H01-00006 | H01-00006-161229-B | 31478 | 29/12/2016 | 23.04 | 0.01 | 99.36 | ERS13431188 | PRJEB55701 | Mixed | D+R- liver recipient | 84 | 80 | 31478 | good | V-GCV or GCV on 1st positive >200 ge/ml. Rx stopped on 2nd negative CMV PCR <200 ge/ml. Subsequent viraemia >3000 ge/ml V-GCV or GCV starts | SOT |  | Adult | No |
|  | H01-00006-170316-B | 12238 | 16/03/2017 | 28.57 | 0.00825 | 99.34 | ERS13431190 | PRJEB55701 |  |  |  |  |  |  |  |  |  |  |  |
| H01-00007 | H01-00007-170116-B | 27769 | 16/01/2017 | 55.15 | 0.00165 | 99.7 | ERS13431191 | PRJEB55701 | Single | D+R- liver recipient | 124 | 97 | 96086 | good | V-GCV or GCV on 1st positive >200 ge/ml. Rx stopped on 2nd negative CMV PCR <200 ge/ml. Subsequent viraemia >3000 ge/ml V-GCV or GCV starts | SOT |  | Adult | No |
|  | H01-00007-170228-B | 53208 | 28/02/2017 | 195.1 | 0.00219 | 99.78 | ERS13431192 | PRJEB55701 |  |  |  |  |  |  |  |  |  |  |  |
|  | H01-00007-170302-B | 96086 | 02/03/2017 | 259.81 | 0.00235 | 99.96 | ERS13431193 | PRJEB55701 |  |  |  |  |  |  |  |  |  |  |  |
|  | H01-00007-170503-B | 14505 | 03/05/2017 | 69.58 | 0.00182 | 99.95 | ERS13431194 | PRJEB55701 |  |  |  |  |  |  |  |  |  |  |  |
| H01-00008 | H01-00008-170314-B | 23677 | 14/03/2017 | 44.47 | 0.00139 | 100 | ERS13431195 | PRJEB55701 | Single | D+R- liver recipient | 31 | 56 | 23677 | good | V-GCV or GCV on 1st positive >200 ge/ml. Rx stopped on 2nd negative CMV PCR <200 ge/ml. Subsequent viraemia >3000 ge/ml V-GCV or GCV starts | SOT |  | Adult | No |
| H01-00009 | H01-00009-170406-B | 48767 | 06/04/2017 | 53.83 | 0.00504 | 98.94 | ERS13431196 | PRJEB55701 | Mixed | D+R- liver recipient | 42 | 56 | 48767 | good | V-GCV or GCV on 1st positive >200 ge/ml. Rx stopped on 2nd negative CMV PCR <200 ge/ml. Subsequent viraemia >3000 ge/ml V-GCV or GCV starts | SOT |  | Adult | No |
| H01-00010 | H01-00010-170523-B | 22223 | 23/05/2017 | 42.34 | 0.00175 | 99.14 | ERS13431198 | PRJEB55701 | Single | D+R- liver recipient | 56 | 91 | 43365 | good | V-GCV or GCV on 1st positive >200 ge/ml. Rx stopped on 2nd negative CMV PCR <200 ge/ml. Subsequent viraemia >3000 ge/ml V-GCV or GCV starts | SOT |  | Adult | No |
|  | H01-00010-170718-B | 29349 | 18/07/2017 | 41.31 | 0.00329 | 99.3 | ERS13431199 | PRJEB55701 |  |  |  |  |  |  |  |  |  |  |  |
| H01-00011 | H01-00011-170621-B | 1327870 | 21/06/2017 | 971.27 | 0.00283 | 100 | ERS13431201 | PRJEB55701 | Single | D+R- liver recipient | 62 | 120 | 1973650 | good | V-GCV or GCV on 1st positive >200 ge/ml. Rx stopped on 2nd negative CMV PCR <200 ge/ml. Subsequent viraemia >3000 ge/ml V-GCV or GCV starts | SOT |  | Adult | No |
|  | H01-00011-170629-B | 18141 | 29/06/2017 | 18 | 0.00105 | 98.69 | ERS13431202 | PRJEB55701 |  |  |  |  |  |  |  |  |  |  |  |
| H01-00012 | H01-00012-170505-B | 4827 | 05/05/2017 | 18.64 | 0.00121 | 97.9 | ERS13431203 | PRJEB55701 | Single | D+R- kidney recipient | 83 | 88 | 116696 | good | V-GCV or GCV on 1st positive >200 ge/ml. Rx stopped on 2nd negative CMV PCR <200 ge/ml. Subsequent viraemia >3000 ge/ml V-GCV or GCV starts | SOT |  | Adult | No |
|  | H01-00012-170518-B | 15679 | 18/05/2017 | 31.74 | 0.00138 | 97.76 | ERS13431204 | PRJEB55701 |  |  |  |  |  |  |  |  |  |  |  |
|  | H01-00012-170606-B | 116696 | 06/06/2017 | 350.17 | 0.00247 | 99.98 | ERS13431205 | PRJEB55701 |  |  |  |  |  |  |  |  |  |  |  |
| H01-00013 | H01-00013-150210-B | 26407 | 10/02/2015 | 77.69 | 0.00389 | 97.22 | ERS13431221 | PRJEB55701 | Single | D+R- liver recipient | 35 | 81 | 26407 | good | V-GCV or GCV on 1st positive >200 ge/ml. Rx stopped on 2nd negative CMV PCR <200 ge/ml. Subsequent viraemia >3000 ge/ml V-GCV or GCV starts | SOT |  | Adult | No |
|  | H01-00013-150331-B | 8011 | 31/03/2015 | 56.15 | 0.00404 | 96.94 | ERS13431222 | PRJEB55701 |  |  |  |  |  |  |  |  |  |  |  |
| H01-00014 | H01-00014-150319-B | 7345 | 19/03/2015 | 37.46 | 0.00877 | 95.86 | ERS13431223 | PRJEB55701 | Mixed | D+R- kidney recipient | 148 | 150 | 37740 | good | V-GCV or GCV on 1st positive >200 ge/ml. Rx stopped on 2nd negative CMV PCR <200 ge/ml. Subsequent viraemia >3000 ge/ml V-GCV or GCV starts | SOT |  | Adult | No |
|  | H01-00014-150507-B | 37740 | 07/05/2015 | 185.61 | 0.00732 | 98.25 | ERS13431225 | PRJEB55701 |  |  |  |  |  |  |  |  |  |  |  |
|  | H01-00014-150810-B | 6689 | 10/08/2015 | 65 | 0.00665 | 97.37 | ERS13431227 | PRJEB55701 |  |  |  |  |  |  |  |  |  |  |  |
| H01-00015 | H01-00015-150423-B | 6987 | 23/04/2015 | 34.88 | 0.0037 | 96.81 | ERS13431229 | PRJEB55701 | Single | D+R- kidney recipient | 49 | 51 | 15053 | good | V-GCV or GCV on 1st positive >200 ge/ml. Rx stopped on 2nd negative CMV PCR <200 ge/ml. Subsequent viraemia >3000 ge/ml V-GCV or GCV starts | SOT |  | Adult | No |
|  | H01-00015-150528-B | 5801 | 28/05/2015 | 41.23 | 0.00397 | 96.75 | ERS13431230 | PRJEB55701 |  |  |  |  |  |  |  |  |  |  |  |
|  | H01-00015-150709-B | 15053 | 09/07/2015 | 61.04 | 0.00413 | 97.85 | ERS13431231 | PRJEB55701 |  |  |  |  |  |  |  |  |  |  |  |
| H01-00016 | H01-00016-150413-B | 106009 | 13/04/2015 | 579.1 | 0.00537 | 99.81 | ERS13431232 | PRJEB55701 | Mixed | D+R- kidney recipient | 273 | 334 | 128711 | good | V-GCV or GCV on 1st positive >200 ge/ml. Rx stopped on 2nd negative CMV PCR <200 ge/ml. Subsequent viraemia >3000 ge/ml V-GCV or GCV starts | SOT |  | Adult | No |
|  | H01-00016-150716-B | 128711 | 16/07/2015 | 881.74 | 0.00415 | 99.98 | ERS13431234 | PRJEB55701 |  |  |  |  |  |  |  |  |  |  |  |
| H01-00017 | H01-00017-150601-B | 184783 | 01/06/2015 | 443.8 | 0.00518 | 99.38 | ERS13431235 | PRJEB55701 | Single | D+R- kidney recipient | 160 | 177 | 184783 | good | V-GCV or GCV on 1st positive >200 ge/ml. Rx stopped on 2nd negative CMV PCR <200 ge/ml. Subsequent viraemia >3000 ge/ml V-GCV or GCV starts | SOT |  | Adult | No |
|  | H01-00017-150716-B | 46459 | 16/07/2015 | 580.87 | 0.00422 | 99.92 | ERS13431236 | PRJEB55701 |  |  |  |  |  |  |  |  |  |  |  |
| H01-00018 | H01-00018-150827-B | 68683 | 27/08/2015 | 246.77 | 0.0053 | 97.94 | ERS13431237 | PRJEB55701 | Single | D+R- kidney recipient | 47 | 93 | 17204 | good | V-GCV or GCV on 1st positive >200 ge/ml. Rx stopped on 2nd negative CMV PCR <200 ge/ml. Subsequent viraemia >3000 ge/ml V-GCV or GCV starts | SOT |  | Adult | No |
|  | H01-00018-150521-B | 2830 | 21/05/2015 | 206.16 | 0.0074 | 98.64 | ERS13431238 | PRJEB55701 |  |  |  |  |  |  |  |  |  |  |  |
| H01-00019 | H01-00019-150928-B | 9490 | 28/09/2015 | 40.88 | 0.00769 | 98.38 | ERS13431239 | PRJEB55701 | Mixed | D+R- kidney recipient | 273 | 334 | 14452 | good | V-GCV or GCV on 1st positive >200 ge/ml. Rx stopped on 2nd negative CMV PCR <200 ge/ml. Subsequent viraemia >3000 ge/ml V-GCV or GCV starts | SOT |  | Adult | No |
| H01-00021 | H01-00021-150313-B | 15010 | 13/03/2015 | 204.45 | 0.00607 | 97.99 | ERS13431241 | PRJEB55701 | Mixed | D+R- liver recipient | 60 | 138 | 15010 | good | V-GCV or GCV on 1st positive >200 ge/ml. Rx stopped on 2nd negative CMV PCR <200 ge/ml. Subsequent viraemia >3000 ge/ml V-GCV or GCV starts | SOT |  | Adult | No |
| H01-00023 | H01-00023-150226-B | 15744 | 26/02/2015 | 59.29 | 0.00398 | 98.84 | ERS13431244 | PRJEB55701 | Single | D+R- kidney recipient | 47 | 106 | 213201 | good | V-GCV or GCV on 1st positive >200 ge/ml. Rx stopped on 2nd negative CMV PCR <200 ge/ml. Subsequent viraemia >3000 ge/ml V-GCV or GCV starts | SOT |  | Adult | No |
|  | H01-00023-150430-B | 213201 | 30/04/2015 | 1060.18 | 0.00387 | 99.92 | ERS13431245 | PRJEB55701 |  |  |  |  |  |  |  |  |  |  |  |
| H01-00024 | H01-00024-150325-B | 26873 | 25/03/2015 | 88.99 | 0.00396 | 98.17 | ERS13431246 | PRJEB55701 | Single | D+R- liver recipient | 30 | 75 | 113837 | good | V-GCV or GCV on 1st positive >200 ge/ml. Rx stopped on 2nd negative CMV PCR <200 ge/ml. Subsequent viraemia >3000 ge/ml V-GCV or GCV starts | SOT |  | Adult | No |
| H01-00025 | H01-00025-141209-B | 5933 | 09/12/2014 | 46.4 | 0.00359 | 98.26 | ERS13431247 | PRJEB55701 | Single | D+R- liver recipient | 45 | 58 | 7917 | good | V-GCV or GCV on 1st positive >200 ge/ml. Rx stopped on 2nd negative CMV PCR <200 ge/ml. Subsequent viraemia >3000 ge/ml V-GCV or GCV starts | SOT |  | Adult | No |
|  | H01-00025-150225-B | 7917 | 25/02/2015 | 70.85 | 0.00486 | 98.82 | ERS13431248 | PRJEB55701 |  |  |  |  |  |  |  |  |  |  |  |
| H01-00026 | H01-00026-141218-B | 14811 | 18/12/2014 | 721.89 | 0.00402 | 99.87 | ERS13431249 | PRJEB55701 | Single | D+R- kidney recipient | 151 | 203 | 337569 | good | V-GCV or GCV on 1st positive >200 ge/ml. Rx stopped on 2nd negative CMV PCR <200 ge/ml. Subsequent viraemia >3000 ge/ml V-GCV or GCV starts | SOT |  | Adult | No |
|  | H01-00026-150102-B | 337569 | 02/01/2015 | 183.28 | 0.00492 | 99.4 | ERS13431250 | PRJEB55701 |  |  |  |  |  |  |  |  |  |  |  |
|  | H01-00026-150202-B | 8306 | 02/02/2015 | 27.84 | 0.0028 | 96.36 | ERS13431251 | PRJEB55701 |  |  |  |  |  |  |  |  |  |  |  |
|  | H01-00026-150604-B | 20389 | 04/06/2015 | 164.49 | 0.00429 | 99.61 | ERS13431253 | PRJEB55701 |  |  |  |  |  |  |  |  |  |  |  |
|  | H01-00026-150723-B | 20335 | 23/07/2015 | 97.07 | 0.00443 | 99.25 | ERS13431254 | PRJEB55701 |  |  |  |  |  |  |  |  |  |  |  |
|  | H01-00026-150910-B | 8499 | 10/09/2015 | 55.6 | 0.004 | 98.28 | ERS13431255 | PRJEB55701 |  |  |  |  |  |  |  |  |  |  |  |
| H01-00027 | H01-00027-160212-B | 8600 | 12/02/2016 | 46.62 | 0.00333 | 96.79 | ERS13431256 | PRJEB55701 | Single | D+R- liver recipient | 23 | 60 | 15115 | good | V-GCV or GCV on 1st positive >200 ge/ml. Rx stopped on 2nd negative CMV PCR <200 ge/ml. Subsequent viraemia >3000 ge/ml V-GCV or GCV starts | SOT |  | Adult | No |
| R01-00014 | R01-00014-191023-B | 88500 | 23/10/2019 | 192.64 | 0.00376 | 99.94 | ERS13431306 | PRJEB55701 | Single | D+R- liver recipient | 145 | 142 | 410000 | poor | V-GCV or GCV on 1st positive >200 ge/ml. Rx stopped on 2nd negative CMV PCR <200 ge/ml. Subsequent viraemia >3000 ge/ml V-GCV or GCV starts | SOT | This the only patient who died in the adult SOT cohort. 42 yo White British female | Adult | No |
|  | R01-00014-190808-B | 11445 | 08/08/2019 | 108.52 | 0.00671 | 99.96 | ERS13431302 | PRJEB55701 |  |  |  |  |  |  |  |  |  |  |  |
|  | R01-00014-191010-B | 512387 | 10/10/2019 | 602.34 | 0.004 | 100 | ERS13431304 | PRJEB55701 |  |  |  |  |  |  |  |  |  |  |  |
|  | R01-00014-190926-B | 39218 | 26/09/2019 | 171.57 | 0.00186 | 100 | ERS13431314 | PRJEB55701 |  |  |  |  |  |  |  |  |  |  |  |
| R01-00016 | R01-00016-180503-B | 23416 | 03/05/2018 | 40.36 | 0.00108 | 99.26 | ERS13431208 | PRJEB55701 | Single | D+R- kidney recipient | 136 | 179 | 150000 | good | V-GCV or GCV on 1st positive >200 ge/ml. Rx stopped on 2nd negative CMV PCR <200 ge/ml. Subsequent viraemia >3000 ge/ml V-GCV or GCV starts | SOT |  | Adult | No |
|  | R01-00016-180621-B | 59342 | 21/06/2018 | 157.8 | 0.00122 | 99.99 | ERS13431209 | PRJEB55701 |  |  |  |  |  |  |  |  |  |  |  |
|  | R01-00016-180816-B | 150000 | 16/08/2018 | 182.78 | 0.00161 | 99.91 | ERS13431210 | PRJEB55701 |  |  |  |  |  |  |  |  |  |  |  |
| R01-00020 | R01-00020-180406-B | 39084 | 06/04/2018 | 197.99 | 0.00142 | 99.79 | ERS13431218 | PRJEB55701 | Single | D+R- liver recipient | 47 | 78 | 300000 | good | V-GCV or GCV on 1st positive >200 ge/ml. Rx stopped on 2nd negative CMV PCR <200 ge/ml. Subsequent viraemia >3000 ge/ml V-GCV or GCV starts | SOT |  | Adult | No |
|  | R01-00020-180412-B | 80784 | 12/04/2018 | 427.73 | 0.00175 | 99.73 | ERS13431219 | PRJEB55701 |  |  |  |  |  |  |  |  |  |  |  |
|  | R01-00020-180711-B | 114070 | 11/07/2018 | 693.81 | 0.00294 | 99.92 | ERS13431220 | PRJEB55701 |  |  |  |  |  |  |  |  |  |  |  |
| R01-00037 | R01-00037-210212-B | 843203 | 12/02/2021 | 1407.55 | 0.00255 | 100 | ERS13431315 | PRJEB55701 | Single | D+R- kidney recipient | 98 | 86 | 930000 | good | V-GCV or GCV on 1st positive >200 ge/ml. Rx stopped on 2nd negative CMV PCR <200 ge/ml. Subsequent viraemia >3000 ge/ml V-GCV or GCV starts | SOT |  | Adult | No |
|  | R01-00037-210222-B | 59002 | 22/02/2021 | 46.41 | 0.00199 | 99.81 | ERS13431316 | PRJEB55701 |  |  |  |  |  |  |  |  |  |  |  |
| R01-00042 | R01-00042-200102-B | 5568 | 02/01/2020 | 34.12 | 0.0018 | 99.81 | ERS13431317 | PRJEB55701 | Mixed | D+R- kidney recipient | 92 | 65 | 55000 | good | V-GCV or GCV on 1st positive >200 ge/ml. Rx stopped on 2nd negative CMV PCR <200 ge/ml. Subsequent viraemia >3000 ge/ml V-GCV or GCV starts | SOT |  | Adult | No |
|  | R01-00042-201205-B | 6664 | 05/12/2020 | 14.4 | 0.01059 | 98.16 | ERS13431319 | PRJEB55701 |  |  |  |  |  |  |  |  |  |  |  |
| R01-00057 | R01-00057-191017-B | 5666 | 17/10/2019 | 37.7 | 0.0014 | 99.72 | ERS13431321 | PRJEB55701 | Single | D+R- kidney recipient | 117 | 139 | 25000 | good | V-GCV or GCV on 1st positive >200 ge/ml. Rx stopped on 2nd negative CMV PCR <200 ge/ml. Subsequent viraemia >3000 ge/ml V-GCV or GCV starts | SOT |  | Adult | No |
|  | R01-00057-191219-B | 10781 | 19/12/2019 | 59.44 | 0.00131 | 99.73 | ERS13431322 | PRJEB55701 |  |  |  |  |  |  |  |  |  |  |  |
|  | R01-00057-200123-B | 10730 | 23/01/2020 | 212.23 | 0.00162 | 99.89 | ERS13431323 | PRJEB55701 |  |  |  |  |  |  |  |  |  |  |  |
|  | R01-00057-200130-B | 5485 | 30/01/2020 | 93.88 | 0.00157 | 100 | ERS13431324 | PRJEB55701 |  |  |  |  |  |  |  |  |  |  |  |
| R01-00060 | R01-00060-191219-B | 2668 | 19/12/2019 | 11.14 | 0.00197 | 99.76 | ERS13431327 | PRJEB55701 | Mixed | D+R- kidney recipient | 125 | 179 | 67000 | good | V-GCV or GCV on 1st positive >200 ge/ml. Rx stopped on 2nd negative CMV PCR <200 ge/ml. Subsequent viraemia >3000 ge/ml V-GCV or GCV starts | SOT |  | Adult | No |
|  | R01-00060-200116-B | 5666 | 16/01/2020 | 12.89 | 0.00169 | 99.58 | ERS13431328 | PRJEB55701 |  |  |  |  |  |  |  |  |  |  |  |
|  | R01-00060-200227-B | 943 | 27/02/2020 | 24.89 | 0.00707 | 99.96 | ERS13431329 | PRJEB55701 |  |  |  |  |  |  |  |  |  |  |  |
| R01-00069 | R01-00069-201022-B | 6542 | 22/10/2020 | 22.1 | 0.00122 | 99.96 | ERS13431331 | PRJEB55701 | Single | D+R- kidney recipient | 154 | 121 | 14000 | good | V-GCV or GCV on 1st positive >200 ge/ml. Rx stopped on 2nd negative CMV PCR <200 ge/ml. Subsequent viraemia >3000 ge/ml V-GCV or GCV starts | SOT |  | Adult | No |
|  | R01-00069-201105-B | 8414 | 05/11/2020 | 101.24 | 0.00172 | 99.94 | ERS13431332 | PRJEB55701 |  |  |  |  |  |  |  |  |  |  |  |
|  | R01-00069-201217-B | 1396 | 17/12/2020 | 19.31 | 0.00153 | 99.69 | ERS13431333 | PRJEB55701 |  |  |  |  |  |  |  |  |  |  |  |
|  | R01-00069-210107-B | 8588 | 07/01/2021 | 29.02 | 0.00144 | 99.97 | ERS13431334 | PRJEB55701 |  |  |  |  |  |  |  |  |  |  |  |
|  | R01-00069-210225-B | 4251 | 25/02/2021 | 19.5 | 0.0015 | 98.81 | ERS13431335 | PRJEB55701 |  |  |  |  |  |  |  |  |  |  |  |
| R01-00079 | R01-00079-191105-B | 3203 | 05/11/2019 | 21.46 | 0.00136 | 99.85 | ERS13431336 | PRJEB55701 | Single | D+R- liver recipient | 69 | 77 | 520000 | good | V-GCV or GCV on 1st positive >200 ge/ml. Rx stopped on 2nd negative CMV PCR <200 ge/ml. Subsequent viraemia >3000 ge/ml V-GCV or GCV starts | SOT |  | Adult | No |
| R01-00117 | R01-00117-210211-B | 5220 | 11/02/2021 | 19.16 | 0.00138 | 99.51 | ERS13431339 | PRJEB55701 | Single | D+R- kidney recipient | 134 | 145 | 9400 | good | V-GCV or GCV on 1st positive >200 ge/ml. Rx stopped on 2nd negative CMV PCR <200 ge/ml. Subsequent viraemia >3000 ge/ml V-GCV or GCV starts | SOT |  | Adult | No |
| R02-00154 | R02-00154-201105-B | 6444 | 05/11/2020 | 33.17 | 0.00821 | 99.88 | ERS13431344 | PRJEB55701 | Mixed | R+ kidney recipient | 143 | 63 | 3600 | good | V-GCV or GCV on 1st positive >200 ge/ml. Rx stopped on 2nd negative CMV PCR <200 ge/ml. Subsequent viraemia >3000 ge/ml V-GCV or GCV starts | SOT |  | Adult | No |
