# Supplementary material for: Persistent Low-Level Variants in a Subset of Viral Genes Are Highly Predictive of Poor Outcome in Immunocompromised Patients With Cytomegalovirus Infection
Source: J Infect Dis. 2024 Jan 5;230(2):e427–36. doi: 10.1093/infdis/jiae001 (PMC11326829; doi:10.1093/infdis/jiae001)
Supplement: jiae001_Supplementary_Data [file jiae001_supplementary_data.zip › Supplementary_material.docx]

**SUPPLEMENTARY FIGURES:**


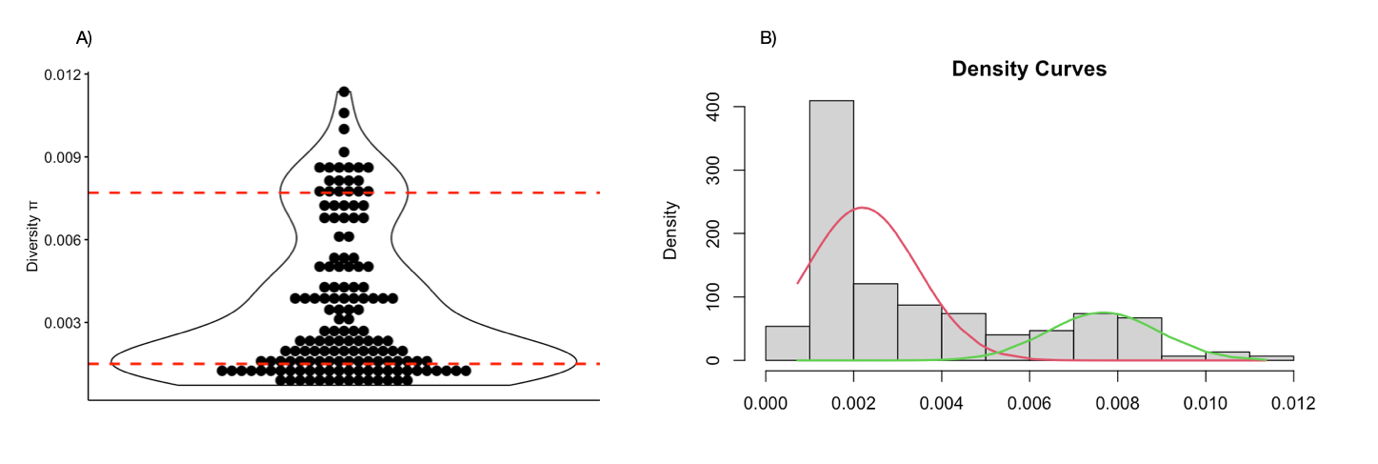


**Supplementary figure 1:** Distribution of diversity values for all samples (A). The distribution of diversity showed bi-modality, each peak fitting a Gaussian distribution crossing at >0.005 (p-value=0.016, first peak/mode estimated at 0.0015 and second peak estimated at 0.0077. Red dashed lines represent the two modes of the bi-modal distribution. The estimated modes were used to create a mixture of Gaussian distributions as shown in the plot (B).

**Supplementary figure 2:** Resistance variants overtime (x-axis) in patients with good (red) and poor (blue) clinical outcome. Variants are considered low-level if the frequency (y-axis) was below 50%.

**
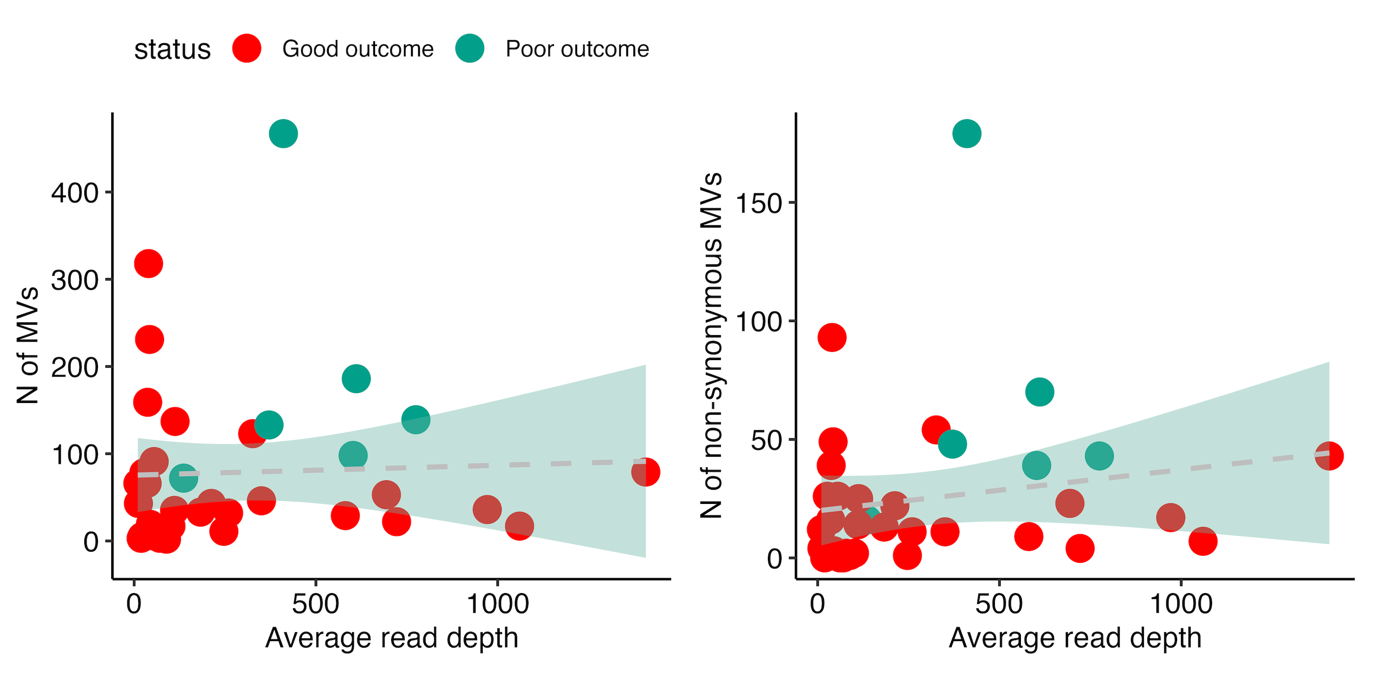
**

**Supplementary figure 3:** Number of minority variants (MVs) and average read depth by sample. Only one sample for patient was selected (based on average read depth). Plot on the left shows the relationship between average read depth and number of all MVs; right panel shows only non-synonymous variants. A trend line with confidence intervals following a linear model was added.


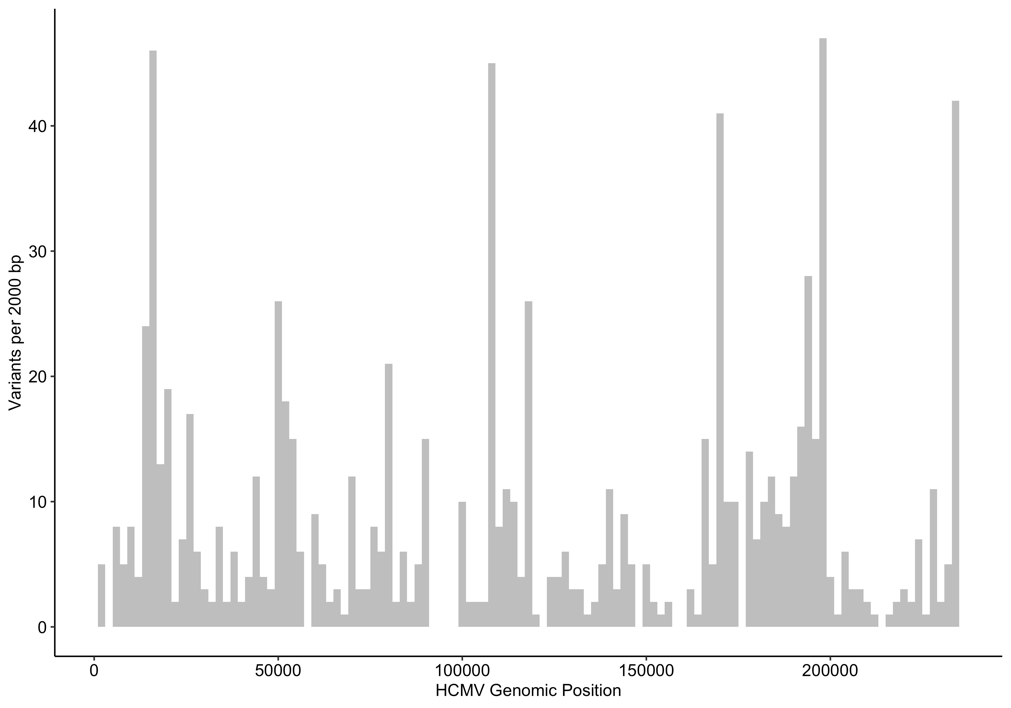


**Supplementary figure 4:** Minority variants distribution across the HCMV genome in all samples


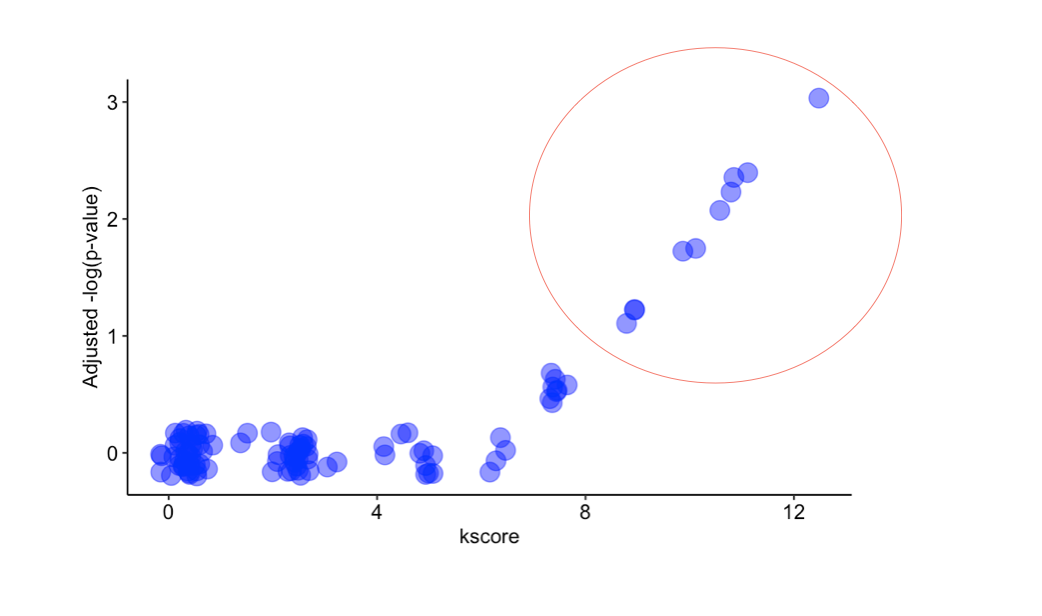


**Supplementary figure 5:** Feature selection results: x-axis shows the k-score and y-axis the -log(p-value) adjusted for multiple testing. We selected the top 10 genes with k-score >8 and p-values <0.005.

**Supplementary figure 6:** ROC curves with confidence intervals (95%) for two predictive models discriminating between samples from patients who died and survivors including all samples from single and mixed infections. AUC for the full model (including MVs in the 10 candidate genes) was 0.91 (red ROC curve). AUC for the drug resistance genes model (including genes UL54 and UL97) was 0.81 (green ROC curve). The two models were significantly different (p-value < 0.001, Anova).


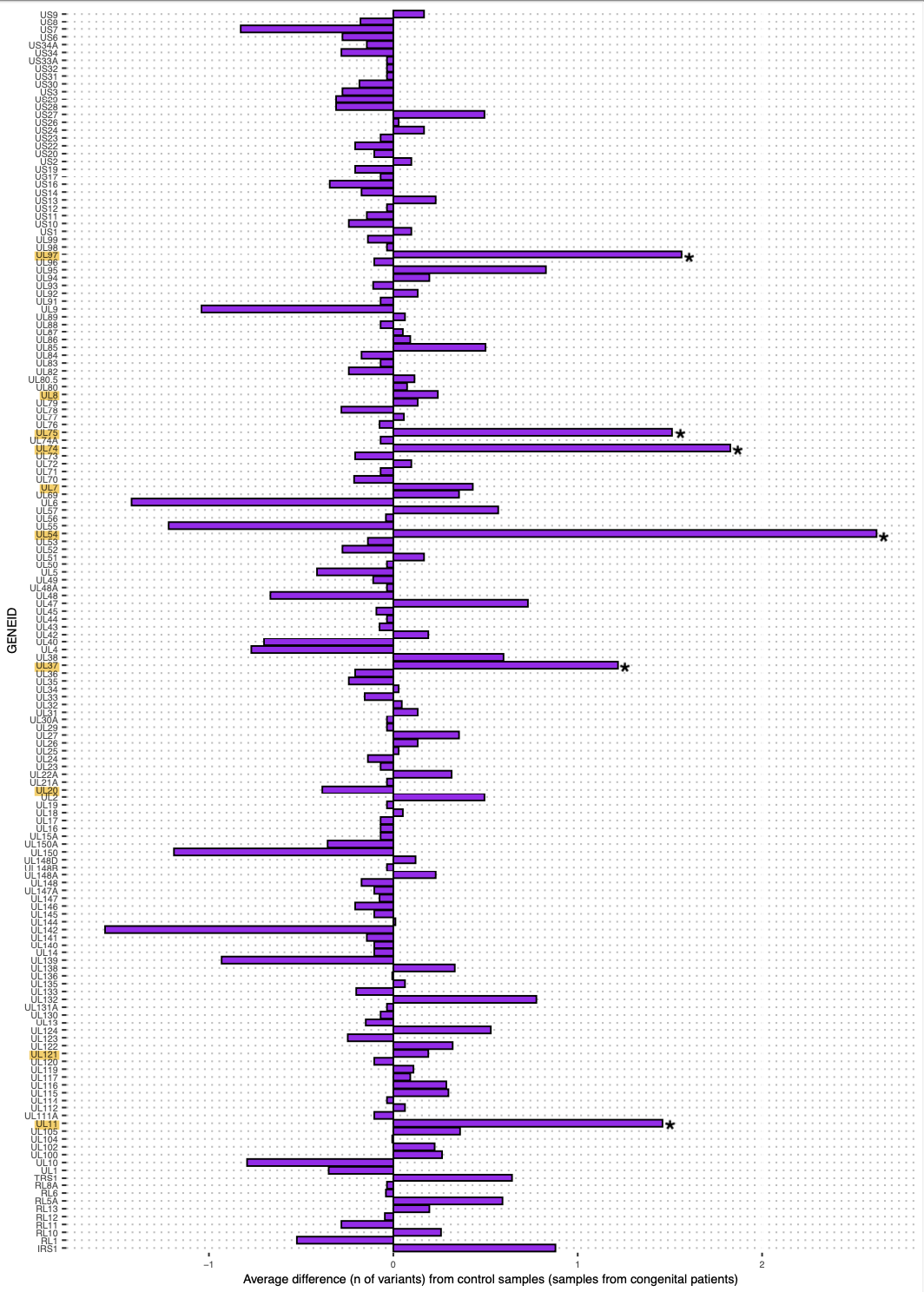


**Supplementary figure 7. Comparison between samples from patients with poor outcome and samples from congenital infections (controls).** The figure shows differences between average number of MVs in samples patients with poor outcome and samples from congenital infections (controls). Only one sample per patient was included in the analysis (the sample with the highest depth). In yellow we underlined genes part of the viral signature. We added an asterisk for genes part of the signature which showed >1 MV difference between groups (6/10). No other gene showed >1 MV.


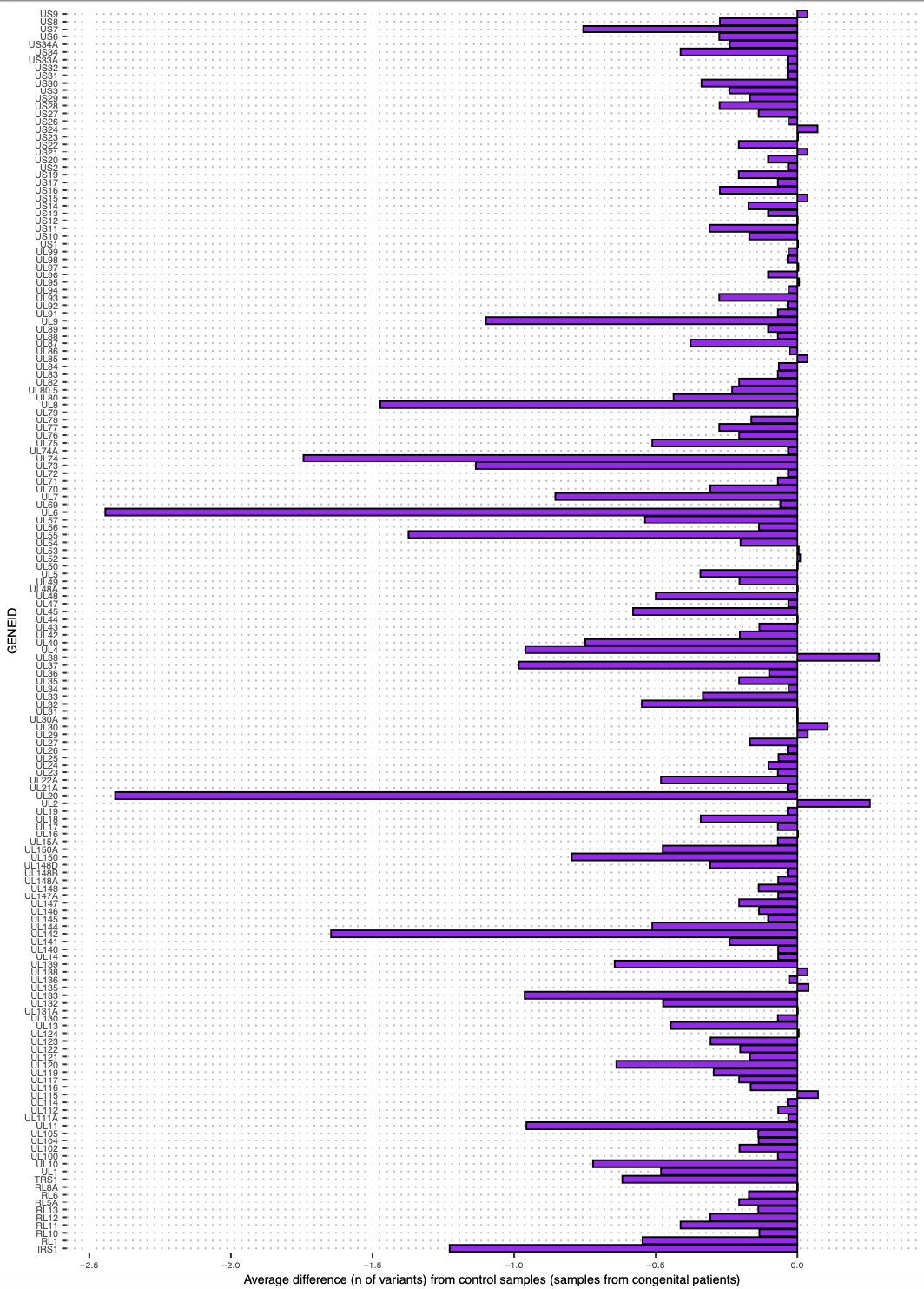


**Supplementary figure 8.** **Comparison between samples from patients with good outcome and samples from congenital infections (controls).** The figure shows differences between average number of MVs in samples patients with good outcome and samples from congenital infections (controls). Only one sample per patient was included in the analysis (the sample with the highest depth). No gene showed greater number of MVs in samples from the good outcome group compared to controls.

**
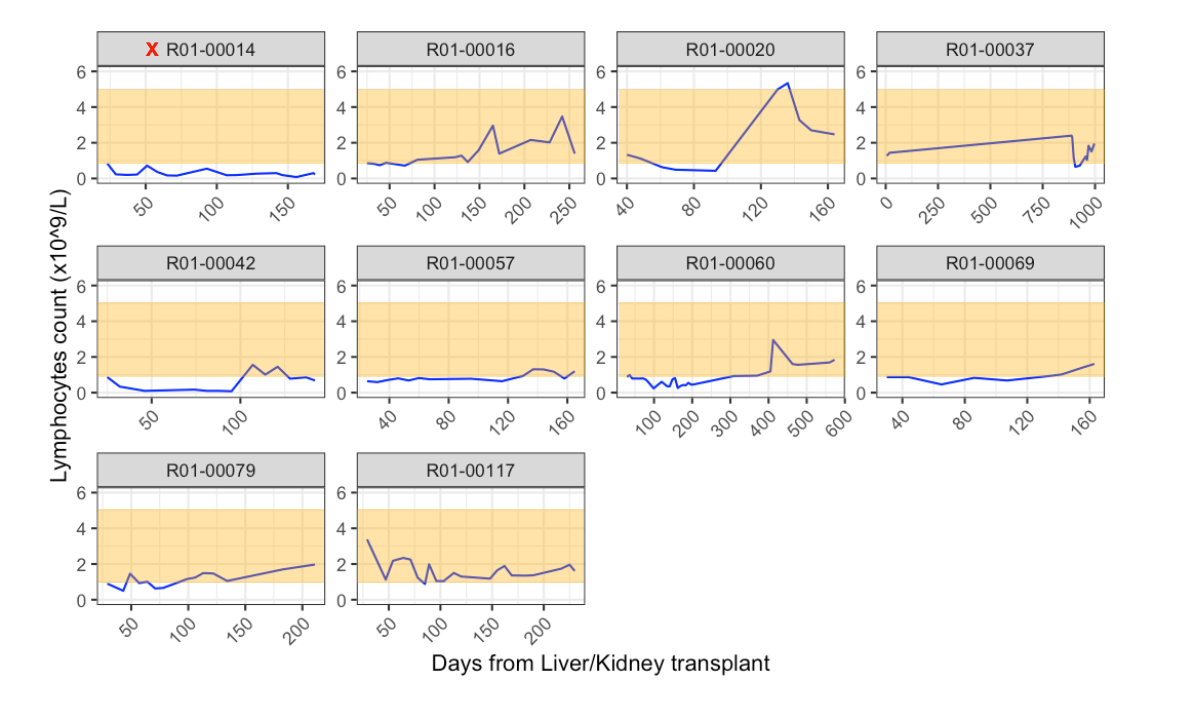
**

**Supplementary figure 9.** Lymphocyte count (per microliter of blood) overtime in a subset of liver/kidney adult patients. The first time point is taken shortly after kidney/liver transplant. In orange we indicated the healthy lymphocyte count range for adults (0.8-5).


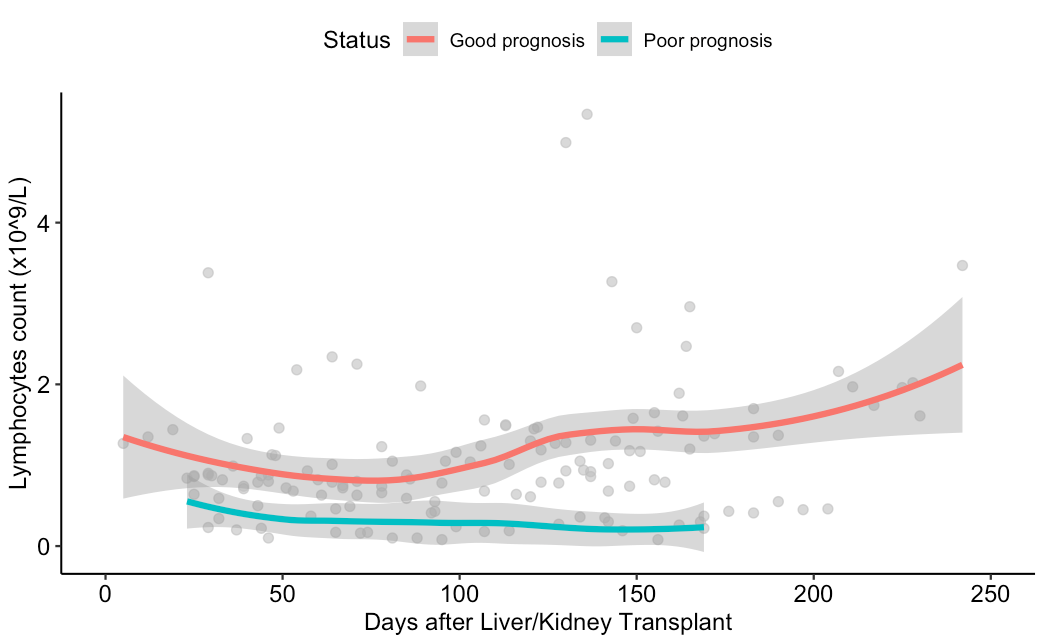


**Figure 10.** Trend lines (smoothed local regression line using loess) for lymphocyte count for good and poor outcome groups in a subset of liver/kidney adult patients. The grey area represents 95% CI. X-axis is restricted to 250 days after transplant.

**SUPPLEMENTARY RESULTS:**

## *Deep-sequencing metrics:*

A total of 141 samples from 51 patients (1-9 samples per patient) were mapped to the HCMV reference strain Merlin genome (NC.006273). Only high-quality samples were included for minority variant screening, that is those with an average sequencing depth of unique reads of ≥10 reads/nt (range of 10x-1407x), and ≥95% coverage of the strain Merlin genome (NC.006273) (Supplementary Database 1).

**SUPPLEMENTARY METHODS:**

*Sequencing:*

Bait Design: To produce the capture probes for hybridisation, biotinylated RNA oligonucleotides (baits) used in the SureSelectXT protocols for CMV were designed in-house. They were synthesised by Agilent Technologies, Santa Clara, California (Agilent Technologies, [2021](https://eur01.safelinks.protection.outlook.com/?url=https%3A%2F%2Fwww.ncbi.nlm.nih.gov%2Fpmc%2Farticles%2FPMC8284422%2F%23B1&data=05%7C01%7Cc.venturini%40ucl.ac.uk%7Cbbe50f13f1c34dffec7a08da9b0156c3%7C1faf88fea9984c5b93c9210a11d9a5c2%7C0%7C0%7C637992727297456316%7CUnknown%7CTWFpbGZsb3d8eyJWIjoiMC4wLjAwMDAiLCJQIjoiV2luMzIiLCJBTiI6Ik1haWwiLCJXVCI6Mn0%3D%7C3000%7C%7C%7C&sdata=TbuOQXBYWvjylOxUs1z6fEfhdH9imhUr4ugW6GPpaJ4%3D&reserved=0)) (available through Agilent’s Community Designs programme:  SSXT CD CMV, part number 5191-6707).

Library prep and sequencing: For whole genome sequencing of CMV, DNA (bulked with male human gDNA (Promega) if required) was sheared using a Covaris E220 focused ultra-sonication system (PIP 75, duty factor 10, cycles per burst 1000). End-repair, non-templated addition of 3′ poly A, adapter ligation, hybridisation, PCR (pre-capture cycles dependent on DNA input and post capture cycles dependent on viral load), and all post-reaction clean-up steps were performed according to either the SureSelectXT Low Input Target Enrichment for Illumina Paired-End Multiplexed Sequencing protocol (version A0) or the SureSelectXT Target Enrichment for Illumina Paired-End Multiplexed Sequencing protocol (version C3) (Agilent Technologies). Quality control steps were performed on the 4200 TapeStation (Agilent Technologies). Samples were sequenced using the Illumina NextSeq 500 platform.

**Statistical analysis**

*Bioinformatics analysis.*

To reliably detect viral variants, we performed careful quality control. The first step involved trimming and quality filtering of the reads, where we selected nucleotides with Phred quality score >20 (1% probability that the variant is due to sequencing error). Reads were trimmed and QC using Trimgalore (<https://www.bioinformatics.babraham.ac.uk/projects/trim_galore/>) and then mapped to the Merlin strain (GenBank Id: NC_006273.2) using BBmap (<https://jgi.doe.gov/data-and-tools/bbtools/bb-tools-user-guide/bbmap-guide/>). We then removed duplicate reads to reduce reads clonality using Picard. Single nucleotide variants present in minor genome populations (minority variants, MVs) were called using Varscan version 2. Parameters used to differentiate true MVs from technical artefacts were as follows: minimum variant allele frequency ≥2%, base call quality of ≥20, and ≥5 unique and independent reads supporting the minor allele. Following this, the ≥2% threshold was only applicable to position with read depth ≥250x (for lower read depth: 50% variant allele frequency for read depth 10x , 10% for read depth 50x and so on).

*Mixed infections.*

Only position with a read depth ≥10x were included for diversity calculation and haplotype reconstruction. Diversity calculations have been described elsewhere (1) and code is available here <https://github.com/ucl-pathgenomics/NucleotideDiversity>. For haplotype reconstruction, we used HaROLD, which uses co-varying variant frequencies in a probabilistic framework. Validation and applications are described here (2) and programs can be found here <https://github.com/ucl-pathgenomics/HaROLD>.

HCMV sample diversity separated into higher and lower diversity groups, with the majority having low within-host diversity. We used the value where the “high diversity” and “low diversity” distributions meet (>0.005) as the cut-off above which infections were considered as potentially mixed (Supplementary figure 1). We then reconstructed haplotypes in 18 patients with suspected mixed infection and identified 14 patients where haplotypes differed by at least 2kbp with the minor haplotypes present at >5% frequency (3) (Figure 1B).

*Feature selection.*

We created a dataset where for each sample we had genes as categorical variables and presence of MVs was indicated as 1/0. Genes with only one mutation in one sample were filtered out. We implemented a gene selection algorithm to evaluate the importance of the presence of low-level variants in a specific gene using Python scikit-learn library (4). Gene selection was done according to the k highest scores (sklearn.feature_selection.SelectKBest with chi-square statistics for classification). Data were split into train/test (70% train, 30% test) 1000 times and, to avoid bias due to longitudinal sampling, we used a Leave-One-Out Cross-Validation (LOOCV) procedure, in particular the shuffle-group-out cross-validation iterator implemented in scikit-learn library (sklearn.model_selection.GroupShuffleSplit) which provides randomized train/test indices to split data according to patient variable. Genes were selected based on 1) top 10 with the highest k-score 2) adjusted -log(p-value) of 1 and k-score of 8.

*Regression model accuracy and probability.*

Code and data to calculate the viral signature score are available <https://github.com/ucl-pathgenomics/HCMV_ViralSignature.git>. This includes the code to calculate HCMV viral signature score for new samples.

To assess the predictive power of using the ten gene viral signature, we compared accuracy for two models: a) a full model including presence/absence of NS MVs in the 10 signature’s genes; and b) a drug resistance gene model, where we only included NS MVs in UL54 and UL97. A generalised logistic model (R function glm, family binomial) was implemented to test the accuracy of the 10-genes model in predicting the clinical outcome. The predict.glm() function in R was employed to generate predictions for each observations in the dataset. Using the ten-genes glm model, we calculated predicted probabilities which represent the model’s estimate of the likelihood that each data point falls into the category associated with an unfavourable clinical outcome.

*Example of R code to run the full model and calculate probabilities.*

$$model\_10g <- glm\left( clinical\_outcome\sim UL97+UL74+UL7+UL8+UL37+UL11+UL20+UL75+UL121+UL54,DF,family=binomial\left( \right) \right)$$

$$scores\_predicted=predict.glm(model\_10g,type=c("response"))$$

$$scores\_predicted\_percentages <- round(scores\_predicted*100)$$

We calculated the True Positive Rate (TPR, sensitivity) and the False Positive Rate (FPR, 1-Specificity). We then built the Receiver Operating Characteristics (ROC) curve and calculated the area under the curve (AUC) which provides an aggregate measure of the performance of the model. ROC curves ( function ‘roc()’) were used to show the sensitivity/specificity for the binary classifier and the area under the curve (AUC) was also calculated.

The results were compared with a model using only the two resistance genes, UL54 and UL97 using an ANOVA test (likelihood-ration, LR).

*Biology of the genes.* Genes were annotated and tested for drug resistance using the R package cmvdrg (5). T cell epitopes for HCMV were extracted from the Immune Epitope Database and Analysis Resource (IEDB). Lymphocytes counts were compared with a mixed effect regression model in R.

1. Cudini J, Roy S, Houldcroft CJ, Bryant JM, Depledge DP, Tutill H, et al. Human cytomegalovirus haplotype reconstruction reveals high diversity due to superinfection and evidence of within-host recombination. Proc Natl Acad Sci. 2019 Mar 19;116(12):5693–8.

2. Venturini C, Pang J, Tamuri AU, Roy S, Atkinson C, Griffiths P, et al. Haplotype assignment of longitudinal viral deep-sequencing data using co-variation of variant frequencies. Virus Evol [Internet]. 2022 Oct 6 [cited 2022 Oct 31]; Available from: https://doi.org/10.1093/ve/veac093

3. Pang J, Slyker JA, Roy S, Bryant J, Atkinson C, Cudini J, et al. Mixed cytomegalovirus genotypes in HIV-positive mothers show compartmentalization and distinct patterns of transmission to infants. Stanley M, Akhmanova A, Ramchandar N, editors. eLife. 2020 Dec 31;9:e63199.

4. Pedregosa F, Varoquaux G, Gramfort A, Michel V, Thirion B, Grisel O, et al. Scikit-learn: Machine Learning in Python. J Mach Learn Res. 2011;12(85):2825–30.

5. Charles OJ, Venturini C, Breuer J. cmvdrg - An R package for Human Cytomegalovirus antiviral Drug Resistance Genotyping [Internet]. bioRxiv; 2020 [cited 2022 Apr 5]. p. 2020.05.15.097907. Available from: https://www.biorxiv.org/content/10.1101/2020.05.15.097907v1
