# Supplementary material for: Persistent Low-Level Variants in a Subset of Viral Genes Are Highly Predictive of Poor Outcome in Immunocompromised Patients With Cytomegalovirus Infection
Source: J Infect Dis. 2024 Jan 5;230(2):e427–36. doi: 10.1093/infdis/jiae001 (PMC11326829; doi:10.1093/infdis/jiae001)
Supplement: jiae001_Supplementary_Data [file jiae001_supplementary_data.zip › Supplementary_tables.docx]

**Supplementary Table 1**. The table shows genes which are part of the viral signature. Genes are ranked by k-score and p-value. Column “All” shows k-score and p-values for analysis including both synonymous and non-synonymous variants.

| **Ranking** | **Gene** | **NS K-score/p-value** | **All K-score/p-value** |
| --- | --- | --- | --- |
| 1 | UL54 | 12.5/4.07e-04 | 3/3.89e-03 |
| 2 | UL20 | 11.2/8.18e-04 | 0.6/4.1e-01 |
| 3 | UL11 | 10.6/1.08e-03 | 11/8.58e-04 |
| 4 | UL8 | 10.6/1.08e-03 | 2/0.15 |
| 5 | UL37 | 10.5/1.18e-03 | 0/1 |
| 6 | UL121 | 10/1.56e-03 | 8/3.89e-03 |
| 7 | UL75 | 10/1.56e-03 | 1.8/1.77e-01 |
| 8 | UL7 | 8.8/2.98e-03 | 18/1.4e-05 |
| 9 | UL97 | 8.8/2.98e-03 | 13/2.57e-04 |
| 10 | UL74 | 8.7/3.09e-03 | 2/1.57e-01 |

**Supplementary Table 2.** Patients with samples classified as “ambiguous”. For each patient, the table shows samples, viral loads and probability scores of a sample to be in the poor outcome group.

| **Patient** | **Longitudinal samples** | | | | | | | | | | |
| --- | --- | --- | --- | --- | --- | --- | --- | --- | --- | --- | --- |
| P16 | Samples | Day 0 | Day 3 | | Day 6 | Day 19 | | Day 20 | Day 52 | | Day 62 |
|  | Viral load (gc/ml) | 803282 | 1016020 | | 553418 | 174148 | | 281838 | 282830 | | 320073 |
|  | Probability | 99% | 28% | | 97% | 40% | | 27% | 76% | | 100% |
| R01-00014 | Samples | Day 91 | | Day 139 | | | Day 153 | | | Day 171 | |
|  | Viral load (gc/ml) | 11445 | | 39218 | | | 512387 | | | 88500 | |
|  | Probability | 100% | | 2% | | | 72% | | | 98% | |
| H01-00017 | Samples | Day 77 | | | | | Day 122 | | | | |
|  | Viral load (gc/ml) | 184783 | | | | | 46459 | | | | |
|  | Probability | 62% | | | | | 0% | | | | |

**Supplementary Table 3.** Samples from amniotic fluid from babies with congenital infections. The table shows ENA accession number, quality control metrics (coverage and average read depth) and within-host diversity.

| **ENA short read accession number** | **Coverage HCMV (NC.006273) genome%** | **Average read depth (after duplicates removal)** | **Within-host diversity (π)** |
| --- | --- | --- | --- |
| ERR3013917 | 100 | 63.31393 | 0.002280368 |
| ERR3013921 | 99.9139 | 124.98773 | 0.001690259 |
| ERR3013922 | 98.9493 | 121.79825 | 0.001576655 |
| ERR3013925 | 99.9363 | 19.0219 | 0.002753646 |
| ERR3013927 | 99.9474 | 42.69843 | 0.001201907 |
| ERR3013928 | 99.9716 | 45.05338 | 0.001145948 |
| ERR3013929 | 99.8557 | 17.3787 | 0.001908297 |
| ERR3013930 | 98.8669 | 160.4097 | 0.001673139 |
| ERR3013935 | 99.9779 | 279.68024 | 0.001555463 |
| ERR3013936 | 100 | 472.08862 | 0.002724851 |
| ERR3013937 | 99.7925 | 15.643 | 0.001641131 |
| ERR3014165 | 99.7933 | 93.04545 | 0.001059753 |
| ERR3014174 | 99.9809 | 226.52142 | 0.001239983 |
| ERR3014176 | 99.9648 | 763.87471 | 0.002257339 |
| ERR3014177 | 99.9559 | 828.6154 | 0.002084565 |
| ERR3014179 | 99.9885 | 985.83931 | 0.001975029 |
| ERR3014180 | 99.9491 | 245.66219 | 0.002580577 |
| ERR3014415 | 99.8595 | 211.9944 | 0.002339532 |
| ERR3014420 | 99.8808 | 593.08297 | 0.002379494 |
| ERR3014421 | 100 | 1300.93251 | 0.001921317 |
| ERR3014423 | 99.9474 | 271.33713 | 0.001696062 |
| ERR3014424 | 100 | 807.44521 | 0.002207601 |
| ERR3014425 | 100 | 291.53812 | 0.002478529 |
| ERR3014426 | 100 | 565.50144 | 0.002361173 |
| ERR3014430 | 100 | 1365.85228 | 0.002189562 |
| ERR3014432 | 100 | 126.67183 | 0.002087194 |
| ERR3014434 | 99.9864 | 1132.82059 | 0.001903497 |
| ERR3014435 | 99.9228 | 908.85106 | 0.002081512 |
| ERR3014901 | 100 | 680.16525 | 0.002400712 |
